# Comprehensive analysis of antibody recognition in convalescent humans from highly pathogenic avian influenza H5N1 infection

**DOI:** 10.1038/ncomms9855

**Published:** 2015-12-04

**Authors:** Teng Zuo, Jianfeng Sun, Guiqin Wang, Liwei Jiang, Yanan Zuo, Danyang Li, Xuanling Shi, Xi Liu, Shilong Fan, Huanhuan Ren, Hongxing Hu, Lina Sun, Boping Zhou, Mifang Liang, Paul Zhou, Xinquan Wang, Linqi Zhang

**Affiliations:** 1 Comprehensive AIDS Research Center, Collaborative Innovation Center for Diagnosis and Treatment of Infectious Diseases, School of Medicine, Tsinghua University, Beijing 100084, China; 2 Ministry of Education Key Laboratory of Protein Science, Center for Structural Biology, School of Life Sciences, Tsinghua University, Beijing 100084, China; 3 Collaborative Innovation Center for Biotherapy, State Key Laboratory of Biotherapy and Cancer Center, West China Hospital, West China Medical School, Sichuan University, Chengdu 610041, China; 4 Unit of Anti-Viral Immunity and Genetic Therapy, Key Laboratory of Molecular Virology and Immunology, Institute Pasteur of Shanghai, Chinese Academy of Sciences, 320 Yueyang Road, Shanghai 200031, China; 5 State Key Laboratory for Infectious Disease Control and Prevention, National Institute for Viral Disease Control and Prevention, China CDC, Beijing 102206, China; 6 Shenzhen Key Laboratory of Pathogen and Immunity, Shenzhen Third People's Hospital, Shenzhen 518112, China

## Abstract

Understanding the mechanism of protective antibody recognition against highly pathogenic avian influenza A virus H5N1 in humans is critical for the development of effective therapies and vaccines. Here we report the crystal structure of three H5-specific human monoclonal antibodies bound to the globular head of hemagglutinin (HA) with distinct epitope specificities, neutralization potencies and breadth. A structural and functional analysis of these epitopes combined with those reported elsewhere identifies four major vulnerable sites on the globular head of H5N1 HA. Chimeric and vulnerable site-specific mutant pseudoviruses are generated to delineate broad neutralization specificities of convalescent sera from two individuals who recovered from the infection with H5N1 virus. Our results show that the four vulnerable sites on the globular head rather than the stem region are the major neutralizing targets, suggesting that during natural H5N1 infection neutralizing antibodies against the globular head work in concert to provide protective antibody-mediated immunity.

## Abstract (web summary)

Unravelling the mechanism of the recognition of the highly pathogenic influenza A viruses by antibodies is important for understanding their protective function. Zuo *et al*. characterise the mechanism of recognition of the H5N1 influenza virus by human monoclonal antibodies isolated from convalescent individuals.

## Graphical abstract

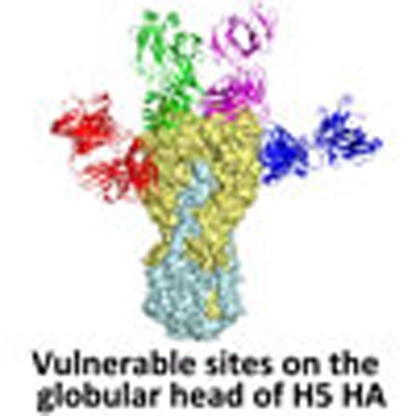

China is one of the hubs for the emergence and dissemination of highly pathogenic avian influenza (HPAI) H5N1 viruses^1^,^2^,^3^. Since its first discovery in a sick goose in Guangdong during the summer of 1996 (ref. 4), HPAI H5N1 has caused frequent outbreaks in domestic poultry farms across the country and resulted in millions of death among chickens, ducks and geese^5^,^6^,^7^. With its unusual pathogenicity, HPAI H5N1 has been exploring other species as hosts across broader geographic frontiers^8^,^9^. Most notable was the concurrent increase in the incidence of human infection as a result of direct exposure to sick or dead poultry and wild birds^7^,^10^,^11^,^12^. The infected human usually manifested severe respiratory symptoms associated with an exceedingly high mortality of more than 60% (refs 11, 12). Mutations in several viral genes have been implicated to increase viral capacity to replicate in a broader range of cell types as well as to attenuate intracellular antiviral immunity^13^. Fortunately, the current HPAI H5N1 strains are rather inefficient in transmission in humans and in other mammals^14^. However, recent ‘gain-of-function' studies showed that by only a few mutations the virus could become airborne transmission in ferrets and guinea pigs^15^,^16^,^17^, raising the serious concerns about its pandemic potential in the near future.

HPAI H5N1 strains isolated from humans worldwide represent a divergent and evolving cluster of quasispecies and can be broadly classified into 10 clades (clades 0–9)^18^. HPAI H5N1 strains identified in China are genetically and antigenically distinct belonging to a previously uncharacterized clade (clade 2.3.4 or Fujian-like) and closely related with those avian isolates in H5N1 genotype Z^6^,^7^,^19^. Antigenic analysis based on hemagglutination inhibition (HI) and microneutralization assays showed reactivity patterns that correlated with the clades or genotypes identified through hemagglutinin (*HA*) gene phylogeny^7^. The effect of hemagglutination inhibition and microneutralization was largely mediated through the interaction between antibodies and viral surface glycoprotein HA. HA is a homotrimeric integral membrane glycoprotein and forms spikes at the surface of the virion. Each monomer is initially synthesized as a full-length precursor HA0 and then cleaved into HA1 and HA2 subunits by host cell proteases. One unique feature of HPAI H5N1 is the presence of polybasic residues at the cleavage site allowing recognition by ubiquitous proteases thereby promoting replication in diverse cell types^7^. The HA1 subunit is primarily responsible for binding the virus to cellular receptor sialic acid, while the HA2 subunit mediates subsequent fusion between the viral envelope and the host cellular endosomal membrane. The HA of HPAI H5N1 has acquired and increased affinity for human α2, 6-linkage receptors and therefore broadened its tropism to greater varieties of cell types^13^, although other factors may also be involved^20^. Structurally, the HA1 subunit demonstrates a globular while the HA2 subunit adopts a hairpin form composed of two antiparallel alpha-helixes^21^,^22^,^23^,^24^.

In influenza infection, neutralizing antibody responses against HA play a critical role in protecting humans from infection and disease progression^25^,^26^,^27^. Most neutralizing antibodies elicited by seasonal influenza infection (H1 and H3) and influenza vaccines target the globular head of HA and are in general strain- or clade-specific due to the highly variable nature of targeted residues^25^,^26^,^27^,^28^,^29^,^30^,^31^. Neutralizing antibodies against the relatively conserved stem region are relatively rare, although some potent and broad stem-specific neutralizing monoclonal antibodies (mAbs) have been identified^29^,^30^,^31^,^32^,^33^,^34^,^35^,^36^. Through systematic analysis of H1- and H3-specific neutralizing mAbs, five antigenic domains (Site A–E) on the globular head of H3 HA and five antigenic sites (Sa, Sb, Ca1, Ca2 and Cb) on the globular head of H1 HA have been well defined and characterized^21^,^23^,^24^. Definition of these antigenic domains/sites has provided structural and functional insights into protective antibody recognition as well as the rationale for optimizing vaccines to match circulating variants. However, our current knowledge on antibody specificity elicited by HPAI H5N1 infection in human remains poorly understood. In particular, we lack structural and functional understanding of antibody recognition and their potential mechanisms of protection during natural infection.

Here we report the systematic characterization of antibody recognition of both neutralizing mAbs and polyclonal sera from two individuals who recovered from HPAI H5N1 infection in China. On the basis of five human neutralizing mAbs isolated earlier by us from the two recovered individuals^37^,^38^, we first solve the crystal structure of three representative mAbs bound to the globular head of HA with distinct epitope specificities, neutralization potencies and breadth. Structural and functional analysis of these epitopes combined with those published elsewhere identifies four major vulnerable sites (VS) on the globular head of HPAI H5N1 HA. More importantly, we showed that convalescent sera contained high levels of neutralizing antibodies targeted to the four VS on the globular head instead of the stem region, suggesting that during natural HPAI H5N1 infection neutralizing antibodies against the globular head worked in concert to provide protective antibody-mediated immunity. We believe that our findings have provided atomic and holistic understanding of protective antibody immunity in HPAI H5N1 recovered individuals and should assist us in the rational design and optimization of therapeutics and vaccines against HPAI H5N1 infection.

## Results

### Binding and neutralization specificity of five human mAbs

We previously reported the isolation and characterization of five human neutralizing mAbs (AVFluIgG01, AVFluIgG03, 65C6, 3C11 and 100F4) from two individuals AH06 and SZ06 who recovered from HPAI H5N1 infection in 2006. AVFluIgG01 and AVFluIgG03 were isolated through panning of an antibody phage library derived from AH06 whereas 65C6, 3C11 and 100F4 were from immortalized memory B cells derived from SZ06 (refs 37, 38). The HPAI H5N1 strains from the two individuals showed >99% homology in their *HA* genes and grouped in the same subclade 2.3.4 within H5N1 (ref. 39). We first studied the neutralization potency and breadth of the five mAbs by testing against a panel of 17 pseudoviruses bearing HA glycoprotein from currently available major clades and subclades of H5N1 (Table 1). 65C6 and 100F4 exhibited the greatest potency and breadth by inhibiting 15 of the 17 representative pseudoviruses with an average inhibitory concentratiion (IC_50_) of 0.012±0.010 and 0.031±0.020 μg ml^−1^, respectively. AVFluIgG01 demonstrated similar breadth but compromised potency with an average IC_50_ of 3.250±8.229 μg ml^−1^. AVFluIgG03 had reasonably good potency with an average IC_50_ of 0.620±1.477 μg ml^−1^ but was only able to neutralize 11 out the 17 pseudoviruses. 3C11, on the other hand, demonstrated the poorest potency with an average IC_50_ of 9.950±18.474 μg mg^−1^ and limited breadth.

**Figure 1:**
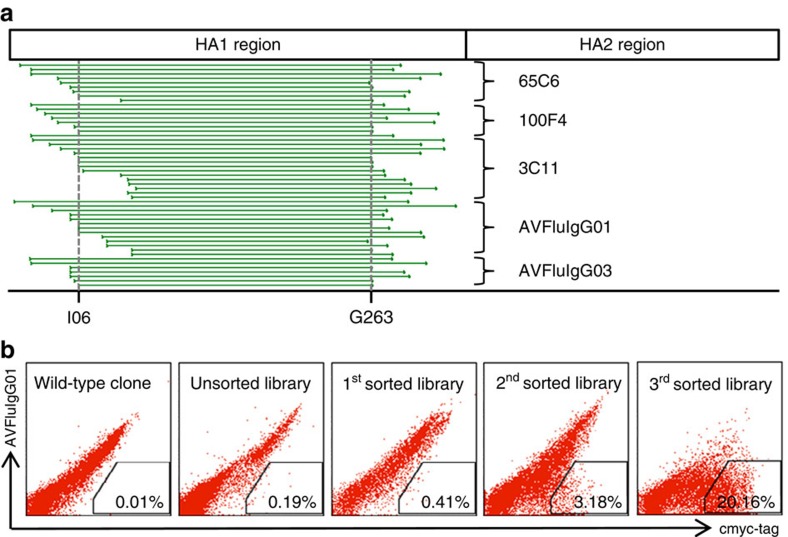
Epitope mapping of five mAbs based on the combinatorial HA library displayed on the surface of yeast. (**a**) Alignment of selected HA fragments by the five mAbs along the A/Anhui/1/2005 full-length HA glycoprotein. Each green horizontal line represents a single selected fragment. The fragment sequence between residues at positions I60 and G263 (F60–263), the minimal stretch shared and recognized by all five mAbs, is indicated by vertical dashed lines. (**b**) Enrichment process for F60–263 mutants that failed to bind to AVFluIgG01. The wild-type clone indicates yeast clone expressing the wild-type F60–263 and the unsorted library refers to the mutated F60–263 library before the selection and sorting process.

To characterize the epitopes recognized by the five human mAbs, we used each of the mAbs to positively select reactive fragments from a combinatorial HA (A/Anhui/1/05) antigen library displayed on the surface of yeast as previously described^40^. Without exception, the selected fragments by all five mAbs fell into the HA1 region, suggesting that the epitopes of these five mAbs must be located within HA1 (Fig. 1a). Analysis of the fragment sequence revealed that residues between I60 and G263 (F60–263) was the minimal stretch shared by all five mAbs. To further map the epitopes, we generated a library of randomly mutated F60–263, incubated with each of the mAbs and sorted for loss of binding to the antibody. Figure 1b illustrates the selection and enrichment process for the mutant F60–263 library that failed to bind to one of the mAbs AVFluIgG01. Increasing proportion of the yeast clones failed to bind to AVFluIgG01 was found from 0.41% for the first sort, to 3.18% for the second and to 20.16% for the third sort. The enriched clones after the third sort were sequenced and the spatial positions of mutated residues were analysed on the three-dimensional structure of A/Anhui/1/05 HA (Supplementary Table 1). Residues buried underneath the HA surface were discarded, while those exposed on the surface were selected to build a panel of mutated pseudoviruses in the context of clade 7 H5N1 A/Beijing/01/03. Among the total of 45 mutant pseudoviruses tested, 14 were found to confer resistance to at least one of the mAbs, although variable impact was noticed for each residue on different mAbs (Table 2). 65C6, 3C11 and AVFluIgG01 shared similar neutralization profile against this panel of pseudoviruses as mutations at similar set of residues such as S126b, K165, R166, Y168 and T171 resulted in a similar trend of decline in their neutralizing activities. Of note, T171A mutation changed residue sequence from NNT to NNA that resulted in the removal of an N-linked glycosylation site (Supplementary Fig. 1). AVFluIgG03 and 100F4, on the other hand, demonstrated different neutralization profiles as each recognized a distinct set of critical residues; E130, S133, K156 and N159 for AVFluIgG03, and D77 and E119 for 100F4 (Table 2). Reduced neutralizing activities appeared to correlate with decreased binding of mAbs to the cell surfaced-expressed HA molecules (Supplementary Fig. 2) but not receptor avidity (Supplementary Fig. 3). Taken together, these results suggested that the epitope specificity of the five mAbs can be broadly classified into three distinct regions on the globular head of HA1 represented by that recognized by 65C6, AVFluIgG03 and 100F4. Of note, four residues (P125H, S126bF, S133L and K156T) demonstrated some degree of cross effect on the mAbs.

### Crystal structures of epitope specificity

To precisely determine epitope specificity, we selected three representative mAbs 65C6, AVFluIgG03 and 100F4 for crystal structural analysis. From the antigen side, we chose an HA1 fragment between residue D55 and E271 (F55–271) largely due to its short length while maintaining the strongest binding to the three mAbs (Supplementary Fig. 4). We first expressed F55–271 and determined its crystal structure at a resolution of 1.5 Å (Supplementary Figs 4 and 5) (Table 3). The F55–271 fragment showed the same three-dimensional fold as that of the A/Anhui/1/05 HA ectodomain (Supplementary Fig. 4)^41^ with an overall root mean square deviation of 0.92 Å for the 213 Cα pairs.

**Figure 2:**
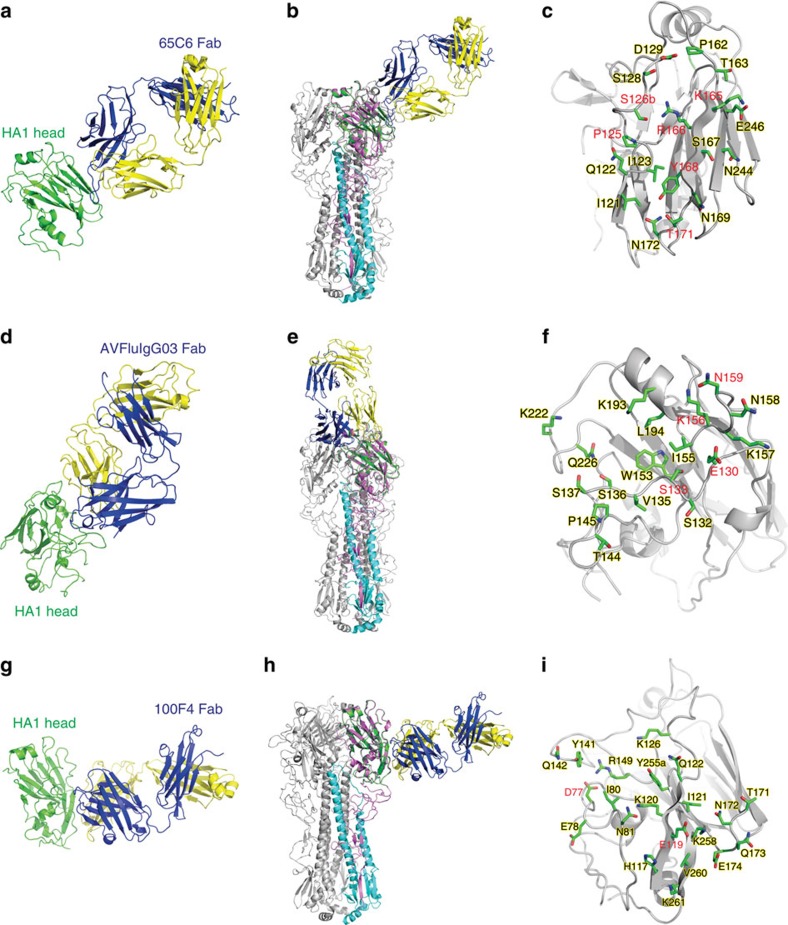
Crystal structural of the globular head complexed with Fab of three representative mAbs. (**a**) The crystal structure of the globular head and 65C6 Fab complex. The head is coloured in green, the heavy chain in blue and light chain in yellow. (**b**) The globular head and 65C6 Fab complex is superimposed onto the A/Anhui/1/05 HA trimer. HA1 is coloured in pink and HA2 in cyan on one of three monomers. (**c**) Closer view of the 65C6 epitope residues shown as green stick. Key residues identified through the yeast surface mutant library are highlighted in red. (**d**–**i**) show the similar contents as that of **a**–**c** except for AVFluIgG03 and 100F4 Fab, respectively.

We then went further to generate the complexes of F55–271 with the Fab of 65C6, AVFluIgG03 or 100F4 and determined their structures at a resolution of 3.0, 3.05 and 2.8 Å, respectively (Table 3 and supplementary Fig. 5). Figure 2a shows the structure of F55–271 in complex with the Fab of 65C6. Superimposition of this complex onto the A/Anhui/1/05 HA trimer showed that 65C6 bound near the membrane-distal end of the HA spike (Fig. 2b), just on the back of the receptor-binding site (RBS). As a typical heavy chain-dominant–binding type, the contacting residues buried 642 Å^2^ surface on HA1, and 662 and 42 Å^2^ surface on the heavy and light chain of 65C6, respectively. Among the three complementarity determining regions (CDRs) H1, H2 and H3, the 22-residue long CDR-H3 was dominant in the binding by contributing 66% of buried surface. The conformational epitope of 65C6 consisted of 18 contacting residues from loop 121–129 and β-strand 162–171 in the receptor binding subdomain (Fig. 2c and Supplementary Table 2), including residues P125, S126b, K165, R166, Y168 and T171 that were identified through the random mutagenesis study (Table 2). The long heavy chain stretched parallel with the β-strand 162–171 and the loop 121–129 of the F55–271, forming hydrophobic and hydrophilic interactions. For example, F104 of CDR-H3 formed hydrophobic interactions with the P125 and R166 residues, D55 of CDR-H2 interacted with K165 through a salt-bridge, and G106 main chain atoms of CDR-H3 formed hydrogen bonds with Y168 and T171 (Supplementary Fig. 6A). Finally, the 18 residues within the epitope demonstrated a substantial degree of sequence conservation with an average of 91.9% homology among the 1,663 sequences of H5N1 HA from various clades and subclades currently available in the database (Supplementary Table 3). The strong potency and breadth demonstrated by 65C6 could therefore be explained by its target conservation compared with those of other mAbs.

Figure 2d shows the structure of F55–271 in complex with the Fab of AVFluIgG03. Superimposition onto the A/Anhui/1/05 HA trimer showed that AVFluIgG03 is bound to the RBS at the membrane-distal end of the HA spike (Fig. 2e). The binding buried 761 Å^2^ surface on HA1, and 514 and 312 Å^2^ surface on the heavy and light chain of AVFluIgG03, respectively. The CDR-H3 of AVFluIgG03 was also 22-residue long and inserted into the receptor-binding pocket, interacting with the 130 loop, 150 loop, 190 helix and 220 loop of HA1 (Supplementary Fig. 6B). The CDR-L1 and CDR-L2 loops further interacted with the 130 and 150 loops, thereby stabilizing the insertion of CDR-H3 into the pocket (Supplementary Fig. 6B). The epitope of AVFluIgG03 consisted of 17 external residues in the RBS including E130, S133, K156 and N159 identified through the random mutagenesis study (Fig. 2f and Supplementary Table 2). This result was in complete agreement with our previous finding that AVFluIgG03 neutralized the virus by directly blocking the attachment of the virus to the host cell^42^. Furthermore, the 18 residues within the epitope had about 84.8% conservation (Supplementary Table 3), significantly less than that of 65C6 (91.9%) and providing explanation for its limited breadth in neutralization (Table 1).

Figure 2g shows the structure of F55–271 in complex with the Fab of 100F4. On the A/Anhui/1/05 HA trimer, the epitope of 100F4 was located right below that of 65C6, across the receptor-binding and vestigial esterase subdomain of HA1 (Fig. 2h). The complex interface occupied 1,041 Å^2^ surface on HA1, and 620 and 475 Å^2^ surface on the heavy and light chain of 100F4, respectively. All six CDRs of 100F4 were involved in the interaction with F55–271, and the epitope consisted of 21 residues (Fig. 2i and Supplementary Table 2). Among these residues, single mutations on D77 and E119 nearly abolished the neutralization activity of 100F4 by disrupting the hydrogen bonds formed between D77 and H54 of CDR-H2 and between E119 and S34 of CDR-L1 (Supplementary Fig. 6C). Finally, the 21 residues had similar degree of conservation (93.2%) compared with that of 65C6 (91.9%) and providing explanation for its strong potency and breadth (Table 1).

### Four major VS on the globular head of H5 HA

**Figure 3:**
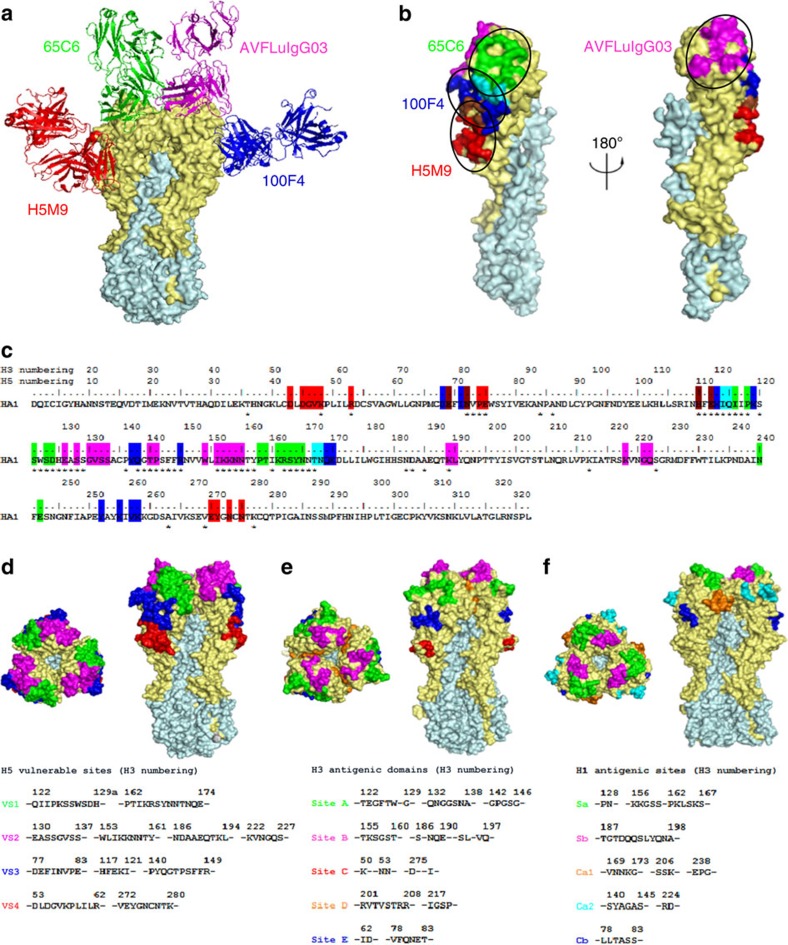
Definition of four major vulnerable sites (VS) on the globular head of H5 HA. (**a**) Structural modeling of 65C6, AVFluIgG03, 100F4 and H5M9 binding to the A/Anhui/1/05 HA trimer. For clarity, binding to two different monomers is shown. HA1 is coloured in light yellow and HA2 in light cyan. The Fabs of 65C6, AVFluIgG03, 100F4 and H5M9 are shown in green, magentas, blue and red, respectively. (**b**) Spatial relationship of 65C6, AVFluIgG03, 100F4 and H5M9 epitopes on the A/Anhui/1/05 HA monomer. The colour for each epitope corresponds to the colour of respective mAb shown in **a**. The overlapping residues between 65C6 and 100F4 epitopes are shown in cyan, and that between 100F4 and H5M9 are shown in brown. (**c**) Sequence comparison of 65C6, AVFluIgG03, 100F4 and H5M9 epitopes with those published elsewhere. Residues within the 65C6, AVFluIgG03, 100F4 and H5M9 epitopes are coloured exactly the same as that for **b**. Residues recognized by other human or mouse mAbs published elsewhere are indicated by asterisks underneath the sequence. (**d**) Structural and spatial modeling of the four VS on the A/Anhui/1/05 HA trimer. Each VS was coloured exactly the same way as that of representing mAbs; VS1-65C6 (green), VS2-AVFluIgG03 (magentas), VS3-100F4 (blue) and VS4-H5M9 (red). (**e**) Structural and spatial modeling of the five antigenic domains on H3 Ha; Site A (green), Site B (magentas), Site C (red), Site D (orange) and Site E (blue). (**f**) Structural and spatial modeling of the five antigenic sites on H1 HA; Sa (green), Sb (magentas), Ca1 (orange), Ca2 (cyan) and Cb (blue).

With atomic structural information on the epitope of the three representative mAbs 65C6, AVFluIgG03 and 100F4, we went further to study their sequence and spatial relationship with those published elsewhere to see whether there were any preferred sites of recognition on H5 HA1 (Supplementary Table 4). There was only one other neutralizing mAb against the globular head of H5 had been structurally characterized and, in this case, a murine-derived mAb, H5M9 (ref. 43). When superimposed onto the A/Anhui/1/05 HA trimer, H5M9 recognized an epitope located on a vestigial esterase subdomain of HA1, distinct from those of 65C6, AVFluIgG03 and 100F4 (Fig. 3a). The H5M9 epitope positioned right below that of 100F4 although there were some overlaps between the two (Fig. 3b). In particular, when the rest of the published epitope sequences were aligned with that of 65C6, AVFluIgG03, 100F4 and H5M9, the majority were found to be either covered in or adjacent to the epitopes of the four mAbs (Fig. 3c). These results indicated that the four structurally defined epitopes of the four mAbs appeared to represent the core recognition of all published human and mouse mAbs. We therefore integrated these epitopes into four major vulnerable sites (VS1–4) on the globular head of HA by taking into account sequence similarity as well as spatial proximity to the four distinct epitopes of 65C6, AVFluIgG03, 100F4 and H5M9, analogous to those antigenic domains/sites identified for H1 and H3 influenza^21^,^23^,^24^. The surface area for each antigenic site was calculated based on the residue composition in the context of three-dimensional structure of A/Anhui/1/05 HA. VS1 was estimated to occupy 1,422 Å^2^ surface on globular head; VS2 1,322 Å^2^; VS3 1,110 Å^2^; and VS4 1,373 Å^2^. The total surface area for VS1–4 constituted ∼52% of the globular head and 30% of A/Anhui/1/05 HA ectodomain.

Figure 3d shows the sequence, location and spatial relationship of the four VS on the A/Anhui/1/05 HA trimer. VS1 (green) and VS2 (purple) were located within the receptor-binding subdomain near the membrane-distal end of the HA spike. VS2 completely covered the RBS, while VS1 occupied the opposite side of the same HA molecule. VS3 positioned at the boundary of the receptor-binding and vestigial esterase subdomain, whereas VS4 was located exclusively in the vestigial esterase subdomain. In general, the distribution pattern of the four VS on H5 HA was quite similar to the antigenic domains/sites of H3 and H1 HAs (Fig. 3e,f). VS1 corresponded to Site A on H3 and Sa on H1, and VS2 to Site B on H3 and Sb on H1. VS3 corresponded to Site E on H3 and Cb on H1, and VS4 to Site C on H3. However, some uniqueness was also found among these sites for H5, H3 and H1. For example, Site D found at the interface between the two different monomers on H3 HA was lacking on H5 HA. While the complete RBS (130 loop, 190 helix and 220 loop) was defined as a part of VS2 on H5 HA, the same RBS was divided into two parts (Site A and B) on H3 HA^23^,^24^. Similarly, loop 77–83 and loop 140–149 within the same VS3 on H5 HA were separated into the two antigenic sites on H1 and H3 HAs^21^,^23^,^24^. Nevertheless, these differences may only reflect availability of different sets of mAbs in defining these sites rather than the genuine differences in antigenicity among H5, H3 and H1 HAs.

### Four VS are targeted in convalescent human

To study the *in vivo* relevance of the four VS defined above, we collected the convalescent sera from two individuals AH06 and SZ06 from whom five human mAbs were initially isolated. We first tested their neutralization potency and breadth against the panel of 17 pseudoviruses bearing HA glycoprotein from currently available major clades and subclades within H5N1 (Table 1). Both serum samples showed high degrees of potency and breadth although variable effect was found for different viral strains. In terms of inhibitory dilution (ID_50_), SZ06 was on average 6,224±4,711, whereas AH06 was 5,663±6,732 dilutions. Among all the pseudoviruses studied, clade 2.3.2.1 (A/common magpie/Hong Kong/5052/2007) was the least sensitive to both sera and thus used later on to study the major targets for broadly neutralizing antibodies in the convalescent sera (see below).

**Figure 4:**
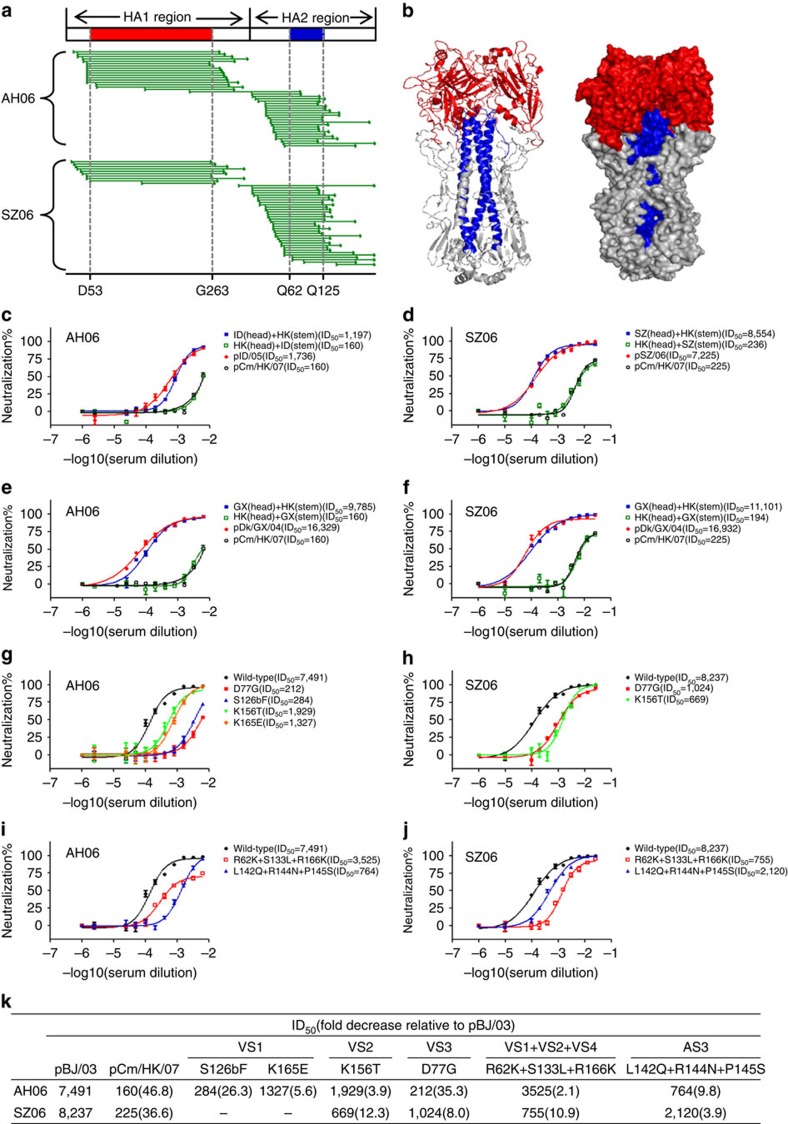
Characterization of antibody recognition in AH06 and SZ06 convalescent sera. (**a**) Alignment of selected fragments along the full-length A/Anhui/1/05 HA. The dominant regions among the selected fragments are located between the residue D53 and G263 in HA1 and G62 and Q125 in HA2, labelled by vertical dashed lines and coloured in red and blue, respectively. (**b**) Structural and spatial modeling of the dominant regions in HA1 (red) and HA2 (blue) on the A/Anhui/1/05 HA trimer. (**c**–**f**) Neutralizing activity of AH06 and SZ06 sera against a panel of wild-type and head/stem chimera pseudoviruses. pID/05 is short for A/Indonesia/5/2005. pCm/HK/07 is short for A/common magpie/HongKong/5052/2007. pSZ/06 is short for A/Shenzhen/406H/2006 whereas pDk/GX/04 is short for A/duck/Guangxi/1378/2004. (**g**–**j**) Neutralizing activity of AH06 and SZ06 sera against a panel of single- and multiple-mutated pseudoviruses. (**k**) Fold changes in ID_50_ of AH06 and SZ06 sera against pseudoviruses with mutations in the four vulnerable sites, compared with the parental strains. pBJ/03 is short for A/Beijing/01/03. For each wild-type and chimera pseudoviruses, neutralization sensitivity was evaluated in at least two independent experiments and the error bars indicate for the standard error of the mean (s.e.m.).

To further delineate the major targets of broad and potent neutralizing activity in the two sera, the yeast library expressing the random fragments of A/Anhui/1/05 HA was incubated with AH06 or SZ06 serum, positively sorted for the reactive fragments by FACS and analysed for their sequences along the full-length HA as previously described^40^. The selected fragments were located in both HA1 and HA2 (Fig. 4a). Those in the HA1 were rather long and similar in length and location to those selected by the five mAbs (Figs 1a and 4a). In contrast, those fragments in HA2 were relatively short. Sequence analysis of selected fragments in HA1 revealed consensus region from residues D53 to G263, virtually overlapping with the entire globular head of HA (Fig. 4b). On the hand other, the consensus sequence in HA2 covered a stretch of residues from Q62 to Q125, corresponding to the long-alpha helix and a previously designed immunogen in inducing protection against divergent influenza subtypes such as H3N2, H1N1 and H5N1 in a mouse model^44^. These results suggested that binding activity of AH06 and SZ06 sera were largely dominated by those targeting to the globular head in HA1 and the long-alpha helix structure in HA2. It needs to be noted, however, that some antigenic regions within HA might have been lost by the fragmentation process during library construction. We have to bear this caveat in mind when interpreting the data.

To further evaluate the relative contribution of the dominant-binding antibody to overall sera neutralization, we generated six HA head/stem chimeric viruses based on four parental strains that were either sensitive (A/Indonesia/5/2005, A/Shenzhen/406H/2006 and A/duck/Guangxi/1378/2004) or resistant (A/common magpie/Hong Kong/5052/2007) to AH06 and SZ06 sera neutralization as described before^45^. For each serum sample, two sets of HA head/stem chimeric viruses were tested to ensure the genuine representation of the results and to minimize the potential confounding effects due to strain selection. As shown in Fig. 4c,e, when the globular head of an insensitive strain (A/common magpie/Hong Kong/5052/2007, labelled in black) was replaced with that from the sensitive strain (A/Indonesia/5/2005 or A/duck/Guangxi/1378/2004, labelled in red), the resultant chimeric viruses (labelled in blue) became sensitive to AH06 sera neutralization. Increases in sensitivity measured by ID_50_ could be as high as 7- to 61-fold, approaching to levels comparable to the parental sensitive strains. In contrast, when the stem region was replaced in the same fashion (labelled in green), negligible changes were found in neutralization sensitivity. Similarly, head replacement from the sensitivity strains (A/Shenzhen/406H/2006 or A/duck/Guangxi/1378/2004, labelled in red) also rendered insensitive strain (A/common magpie/Hong Kong/5052/2007, labelled in black) sensitive to SZ06 sera neutralization (Fig. 4d,f). In terms of ID_50_, it increased approximately 38- to 49-fold and close to that of parental sensitive viruses. Replacement of stem region, however, had little if not at all impact on viral sensitivity to SZ06 sera neutralization (labelled in green). These results indicated that the globular head, not the stem region, was the dominant region recognized by the broad and potent neutralizing antibodies in convalescent sera from AH06 and SZ06.

Next, we asked whether the four VS identified on the globular head were preferentially recognized by the convalescent sera. To this end, we tested serum-neutralizing activity against a panel of VS-specific mutant pseudoviruses with either single or multiple residue substitutions. We reasoned that if a pseudovirus with mutations in any of VS became less susceptible to serum neutralization compared with the parental strain, the serum should contain the neutralizing antibodies targeting the corresponding site. We first tested the neutralizing activity of AH06 and SZ06 serum against the 14 mutant pseudoviruses used to map the epitope specificity of the five mAbs (Table 2). As shown in Fig. 4g,k, a single substitution S126bF within VS1 and D77G within VS3 rendered the pseudoviruses about 26- and 35-fold less sensitive to AH06 serum compared with the parental strain. To a lesser extent, single substitutions at K165E within VS1 and K156T within VS2 also reduced viral sensitivity to AH06 serum about 6- and 4-fold, respectively. For SZ06 serum, K156T within VS2, and D77G within VS3 were able to reduce viral sensitivity about 12- and 8-fold, respectively (Fig. 4h,k). In addition, we investigated whether naturally occurring variations within the four VS could also change the viral sensitivity to AH06 and SZ06 serum neutralization. Analysis of HA sequences from the 17 pseudoviruses representing the major clades and subclades within H5N1 revealed genetic polymorphisms in VS1–4 that were potentially associated with the altered sensitivity to serum neutralization (Supplementary Table 5–8). We have, therefore, constructed pseudoviruses with selected natural mutations and tested their changes to AH06 and SZ06 serum neutralization (Fig. 4i,j,k). Specifically, the triple-mutant R62K+S133L+R166K located in VS1, VS2 and VS4 combined resulted in 2- and 11-fold more resistance to AH06 and SZ06 serum neutralization, respectively. Another triple-mutant L142Q+R144N+P145S located in VS3-rendered pseudovirus about 10- and 4-fold more resistance to AH06 and SZ06 serum neutralization, respectively. It needs to be noted that L142Q+R144N+P145S mutations resulted in addition of an N-linked glycosylation site and might have played a role in conferring resistance to AH06 and SZ06 sera (Supplementary Fig. 1). Taken together, these results indicated that the four VS defined on the globular head were the major targets for neutralizing antibodies in the convalescent sera.

## Discussion

We report here the structural and functional analysis of antibody responses in two convalescent individuals in China from clade 2.3.4 HPAI H5N1 infection. The uniqueness of our study lies in the comprehensive approach to characterize and connect monoclonal and polyclonal antibody responses from the same individuals to provide more complete understanding of protective antibody response against HPAI H5N1 infection in human. At the mAb level, we provided atomic details of three epitopes recognized by three representative human-neutralizing mAbs out of the five we isolated earlier^37^,^38^. Built on the epitopes identified here and integrating with those reported elsewhere, we were able to define antibody recognition into four major VS on the globular head of HA, analogous to those identified antigenic sites/domains for H1 and H3 influenza^21^,^23^,^24^. At the polyclonal antibody level, the binding activities were clearly directed to both the globular head in HA1 and the long-alpha helix of HA2. The majority of neutralizing antibody response, however, was clearly mediated by globular head-targeted antibodies. Within the globular head, the four VS were indeed recognized in a fashion similar to that by the mAbs isolated from the same patients. Our study has, therefore, provided solid evidence from both an atomic and a holistic perspective that the globular head rather than the stem are the major neutralizing targets and likely provide protective antibody-mediated immunity during nature infection.

It has been well argued that the HA glycoprotein is a moving target in the face of antibody response. Antigenic drift frequently occurs on the globular head of HA during infection and transmission to facilitate viral escape from antibody neutralization^25^,^26^,^27^,^46^. A major question remains on how the antibody response copes with such high degree of antigenic drift to provide protective immunity. To this end, we have compared the specificity and mode of recognition by antibodies against various HA subtypes by docking all the HA-antibody structures solved to date onto A/Anhui/1/05 HA (Supplementary Fig. 7). We found that antibody recognition was directed to both HA1 and HA2. While all stem antibodies converged into one major site, head antibodies tended to approach HA in four major ones and each of which correlated with one of the four major ‘vulnerable sites' defined in the current study. As these targets demonstrated variable degree of genetic polymorphism, it is expected that different mAb may offer different degree of protective potential. In fact, this hypothesis is strongly supported in the experimental animals and implicated in human studies^33^,^34^,^35^,^37^,^38^,^42^,^47^. However, in a given infected individual, mAbs targeted at different sites were expected to work in concert to minimize or prevent viral escape from neutralization. Only through collective effort, the antibody response could provide timely and long-lasting protective immunity against the highly drifted and moving target of influenza. On the flip side, it also implies that viruses would have to acquire mutations in all major epitopes before becoming fully capable of escaping from antibody recognition *in vivo*. Indeed, such collective mutations in multiple antigenic sites were found during the turnover of different influenza epidemics^23^.

The structural insights into the antibody specificity also indicated distinct mechanisms of neutralization among the different mAbs. As epitope of AVFluIgG03 completely overlapped with RBS, the neutralization mechanism of AVFluIgG03 would be expected to directly block virus binding. In fact, we compared the structural similarity between the recognition of the RBS by AVFluIg03 and other mAbs (CH65, C05 and S139/1) targeting the same site^48^,^49^,^50^ and found all these mAbs interacting with the RBS through receptor mimicry, although the actual interacting residues differed from antibody to antibody (Supplementary Fig. 8). However, S139/1, previously showed to have broad reactivity against multiple HA subtypes, failed to have detectable activity against the H5 HA studied here (Supplementary Fig. 9). In contrast, epitopes of 65C6 and 100F4 were located away from RBS and their major mode of action would therefore be expected at steps after receptor binding. In fact, this hypothesis was in complete agreement with our mechanistic studies on these mAbs reported earlier^42^. Similarly, as the epitope of murine mAb H5M9 and stem antibodies was located further to the membrane-proximal end of H5 HA, the major mode of their neutralization would be expected to act on steps after the virus binding to its receptor, although other modes of action could not be fully excluded^43^. Taken together, the collective nature of mAbs on neutralization, and by inference the polyclonal antibodies, was not only reflected on the recognition of distinct epitopes *per se* but also on inhibiting multiple steps during viral entry. Temporal and spatial coordination to block multiple steps during entry would be expected to provide the best protective immunity possible against the moving target of influenza.

We believe that the results from our study have direct implications for the development and optimization of vaccines against HPAI H5N1 and other influenza viruses in general. Current influenza vaccines based on the inactivated or live attenuated influenza variants aim to elicit neutralizing antibody responses against the major antigenic sites on the globular head of HA. In most cases, however, the vaccines have relatively poor immunogenicity reflected by relative low titres of neutralizing antibodies induced in vaccinated individuals particularly in the elderly^26^,^51^,^52^. With the help of novel adjuvant such as MF59, significant increases in neutralizing antibody titres were found largely due to expanded and enhanced antibody recognition from HA2 to the globular head of HA1 (refs 26, 53, 54). This finding was supportive of our profiling studies on convalescent sera from the two individuals where binding antibodies were found to both HA1 and HA2, while the major neutralizing antibodies were confined to the globular head of HA1 (Fig. 4). Although HA2 sequences are more conserved and are currently being developed for ‘universal' influenza vaccines, in some cases, vaccine-induced HA2-specific antibodies were also shown to promote virus fusion and enhance influenza respiratory disease^55^. These findings indicate that, in both vaccination and natural infection, HA2 may exert a dominant-negative effect on the induction of neutralizing antibodies against the globular head of HA1. Future vaccine strategies targeting at HA2 would need to identify and eliminate these adverse elements. On the other hand, vaccine strategies built on the structurally intact globular head of HA1 would be expected to have stronger immunogenicity in inducing protective neutralizing antibodies than the current forms of influenza vaccines based on intact HA glycoprotein. While some preliminary results supported these hypotheses^56^,^57^,^58^, definitive conclusions would require more thorough and systematic investigations preferably in human studies.

## Methods

### Ethics statement

The study was carried out in strict accordance with Chinese rules and regulations for the protection of human subjects. Institutional Ethical Committee at Shenzhen Third People's Hospital, Shenzhen, China approved the study. The study subjects provided the written informed consents for the research use of their blood samples.

### Convalescent serum samples and mAbs

The convalescent blood samples were obtained from two individuals AH06 and SZ06 ∼4 and 6 months after onset of disease, respectively. HPAI H5N1 isolated from both individuals fell into the same subclade 2.3.4 within the H5N1 (refs 7, 39). AH06 was a 26-year-old female from Anhui province who became infected in early February 2006 following contact with diseased poultry^7^. SZ06 was a previously healthy 31-year-old male van driver from Shengzhen in Guangdong province. He was confirmed HPAI H5N1 infected through a reverse transcriptase–PCR assay and H5N1 virus was isolated from a tracheal aspirate in June 2006 (ref. 7). During the first 2 weeks after the onset of symptom, SZ06 was treated with oseltamivir and three 200-ml transfusion of AH06 convalescent plasma at the Shenzhen Third People's Hospital, Shenzhen, China^39^. mAbs AVFulgG01 and AVFulgG03 were isolated through panning of a Fab antibody phage library derived from AH06, whereas 65C6, 3C11 and 100F4 were screened from memory B cells derived from SZ06 (refs 37, 38).

### Production and purification of human mAbs

The heavy and light chain genes of each mAb were cloned into the antibody expression vector containing the constant regions of human immunoglobulin G1 (IgG1). Full-length IgG1 were expressed by transient transfection of 293 T cells originally obtained from ATCC, purified by affinity chromatography using Protein A agarose (Pierce,Thermo), and titered by BCA Protein Assay Kit (Thermo Scientific).

### Epitope mapping of mAbs by yeast surface display

Epitope mapping of the five mAbs was carried out at both domain and residue levels based on the yeast surface display library as previously reported^40^. Briefly, the combinatorial fragment library was stained with each of the mAb and positively sorted by FACS using Aria II (BD, USA). The sorted positive yeast clones displaying the respective antigenic fragments were harvested and the plasmids encoding the corresponding fragments were extracted and subjected to sequencing and sequence analysis. Once the domain level of recognition had been identified, the corresponding gene fragment was used to generate additional library using error-prone PCR. Specifically, PCR-amplified random mutagenesis library was gel purified and cloned into yeast surface display vector. The cloned products were then transformed into competent yeast cell line EBY100 using electroporation. The yeast library was induced and incubated with each of the five mAbs and negatively sorted for loss of binding. Anti-cmyc tag antibody was used as a control.

### Generation of wild-type and mutated pseudoviruses

Pseudoviruses bearing the wild-type HA glycoprotein from currently available major clades and subclades of H5N1 were constructed and were previously reported^37^,^42^. Pseudoviruses with single or multiple mutations were generated by site-directed mutagenesis in the context of clade 7 H5N1 A/Beijing/01/03. Site-directed mutagenesis was conducted according to the instruction manual of QuikChange II Site-Directed Mutagenesis Kit (Stratagene Catalogue #200523). Head/stem chimeric pseudoviruses were prepared by overlapping PCR between the sensitive (A/Indonesia/5/2005, A/Shenzhen/406H/2006 and A/duck/Guangxi/1378/2004) and the resistant (A/common magpie/Hong Kong/5052/2007) strains to AH06 and SZ06 serum neutralization. In the head/stem chimeras, the head region consisted of HA1 segment between residues 52 and 277, and the stem included the HA2 and two segments from HA1 between residues 1–41 and 275–329. The numbering system used in this manuscript was based on H3 viruses. To improve the production of pseudoviruses, the codon-optimized HA were used throughout the experiments.

### Pseudovirus-based neutralization assay

Pseudovirus-based neutralization assay was conducted as previously described^37^,^42^. Briefly, MDCK cells (3 × 10^3^ cells per well) were seeded onto 96-well plate in complete DMEM overnight. Serially, twofold dilutions of each mAb or serum sample were incubated with pseudovirus at the final volume of 100 μl at 37 °C for 1 h before added to the seeded MDCK. The relative luciferase activity was measured after 48 h by the BrightGlo Luciferase assay according to the manufacturer's instruction (Promega, USA). Titration curves were generated using sigmoid dose response of nonlinear fit from GraphPad, and inhibitory concentration (IC_50_) and inhibitory dilution (ID_50_) values were determined where 50% reduction of relative luciferase activity was identified.

### Production of globular head and Fab and their complexes

The globular head of H5 HA was expressed using the Bac-to-Bac baculovirus expression system (Invitrogen). The DNA fragment corresponding to residues D55 and E271 (F55-271) of the A/Anhui/1/2005 HA was inserted into a pFastBac dual vector (Invitrogen), with an N-terminal gp67 signal peptide to facilitate secretion and a C-terminal 6-His tag for purification. The recombinant bacmids were transfected into Sf9 insect cells using Cellfectin II Reagent (Invitrogen). Secreted globular head protein in the cell supernatants was captured by nickel-nitrilotriacetic acid (Ni-NTA) resin, eluted with 500 mM imidazole, and then further purified by size exclusion chromatography on a Superdex 200 column (GE Health Care). The Fab fragment was produced by digesting full-length 65C6, 100F4 and AVFluIgG03 IgG with papain for 5 h using Pierce Fab Preparation Kit (Thermo #44985) and further purified by gel filtration (Superdex 200 column, GE Health Care). The globular head and Fab complexes were generated by mixing the two components together, incubated on ice for 1 h and purified by size exclusion chromatography on a Superdex 200 column (GE Health Care).

### Crystallization of globular head alone and complex with Fab

Purified globular head monomer was concentrated to 20 mg ml^−1^ in HEPES-Buffered Saline (HBS) buffer (10 mM HEPES pH 7.2, 150 mM NaCl) for crystallization. Crystals were successfully grown at room temperature by vapour diffusion in sitting drops composed of equal volumes of protein solution and reservoir solution containing 0.08 M magnesium acetate tetrahydrate, 0.05 M sodium cacodylate trihydrate pH6.5, and 30% w/v polyethylene glycol 4,000. The globular head and Fab complex was collected and concentrated to ∼20 mg ml^−1^ in HBS buffer (10 mM HEPES, pH 7.2, 150 mM NaCl) for crystallization. Crystals were successfully grown using sitting drop vapour diffusion method as that for the globular head monomer. The crystals of globular head and 65C6 Fab complex were grown at the reservoir solution condition containing 2% w/v polyethylene glycol 400, 0.1 M imidazole pH 7.0, 24% w/v polyethylene glycol monomethyl ether 5000. The crystals of globular head and 100F4 Fab complex were grown at the reservoir solution condition containing 4.0 M potassium formate, Tris propane pH 9.0, 2% w/v polyethylene glycol monomethyl ether 2000. The crystals of globular head and AVFluIgG03 Fab complex were grown at the reservoir solution condition containing 0.2 M potassium sodium tartrate tetrahydrate, 20% w/v polyethylene glycol 3350. Crystals were frozen in liquid nitrogen with cryoprotectant (well solution plus 20% (v/v) glycol) before data collection. Diffraction data were collected at the BL17U beam line of the Shanghai Synchrotron Research Facility and processed with HKL2000 (ref. 59). All data collection and processing statistics are listed in Table 3.

### Structural determination and refinement

The structure of globular head was determined by molecular replacement with the program PHASER^60^. The search model is the globular head region of H5 of an avian influenza virus A/Indonesia/5/2005 (Protein Data Bank ID code 4K62). Three Fab-H5 globular head complex structures were also determined by molecular replacement. The search models are the refined H5 globular head structure and the structures of variable and constant domain of heavy and light chains available in the Protein Data Bank with the highest sequence identities. Iterative refinement with the program PHENIX and model building with the program COOT were performed to complete the structural refinement^61^. Structure validation was performed with the program PROCHECK^62^, and all structural figures were made with PYMOL^63^. All structural refinement statistics are listed in Table 3.

## Additional information

**Accession codes:** Atomic coordinates and structure factors for the crystal structures H5 globular head and its complexes with Fab of 65C6, AVFluIgG03 and 100F4 have been deposited in the Protein Data Bank with the accession codes 5DUT, 5DUM, 5DUP and 5DUR, respectively.

**How to cite this article:** Zuo, T. *et al*. Comprehensive analysis of antibody recognition in convalescent humans from highly pathogenic avian influenza H5N1 infection. *Nat. Commun.* 6:8855 doi: 10.1038/ncomms9855 (2015).

## Supplementary Material

Supplementary Information Supplementary Figures 1-9, Supplementary Tables 1-8 and Supplementary References

## Figures and Tables

**Table 1:** Neutralization breadths and potencies of the five mAbs and convalescent sera.

**Virus strain**	**Clades**	**IC**_**50**_ **(μg ml**^**−1**^)					**ID**_**50**_**dilutions**	
		**65C6**	**100F4**	**3C11**	**AVFluIgG01**	**AVFluIgG03**	**SZ06**	**AH06**
A/Hong Kong/156/1997	0	0.005	0.05	0.018	0.066	0.008	3,202	664
A/Thailand/(KAN-1)/2004	1	0.029	0.067	ND	0.045	ND	6,231	1,957
A/Indonesia/5/2005	2.1.3.2	0.004	0.013	0.167	0.052	4.752	8,613	1,736
A/Turkey/65596/2006	2.2.1	0.002	0.004	0.118	0.006	1.861	8,598	2,562
A/common magpie/Hong Kong/5052/2007	2.3.2.1	0.034	0.066	ND	30.25	ND	225	160
A/Shenzhen/406H/2006	2.3.4	0.02	0.052	2.631	0.012	0.002	7,225	2,681
A/chicken/Guangxi/12/2004	2.4	0.005	0.015	6.85	12.667	ND	2,527	2,630
A/chicken/Korea/es/2003	2.5	0.005	0.016	1.047	0.016	ND	4,270	5,010
A/silky chicken/Hong Kong/SF189/2001	3	0.011	0.022	5.183	0.008	0.069	13,394	7,800
A/goose/Guiyang/337/2006	4	0.025	0.019	ND	5.556	ND	912	307
A/duck/Guangxi/1378/2004	5	0.005	0.021	0.379	0.019	0.009	16,932	16,329
A/blackbird/Hunan/1/2004	6	0.007	0.033	29.422	0.015	0.004	3,975	10,537
A/Beijing/01/2003	7	0.006	0.037	0.14	0.008	0.003	8,237	7,491
A/Chicken/Vietnam/NCVD-03/08	7.1	ND	ND	ND	ND	0.069	2,235	1,392
A/Chicken/Vietnam /NCVD-16/08	7.1	ND	ND	ND	ND	0.03	3,022	951
A/chicken/Henan/16/2004	8	0.014	0.036	11.257	0.026	ND	3,447	25,381
A/goose/Shantou/1621/2005	9	0.007	0.011	62.192	0.002	0.018	12,768	8,681
VSVG		ND	ND	ND	ND	ND	ND	ND
Average		0.012	0.031	9.950	3.250	0.620	6,224	5,663
s.d.		0.010	0.020	18.474	8.229	1.477	4,711	6,732

mAb, monoclonal antibody; ND, not detected.

**Table 2:** Impact of single residue mutation on pseudovirus sensitivity to antibody neutralization.

**Mutation**	**IC**_**50**_ **(folds increase relative to wild type)**				
	**65C6**	**3C11**	**AVFluIgG01**	**AVFluIgG03**	**100F4**
I124T	−	−	>50	−	−
P125H	−	>50	>50	−	2.7
S126bY	2.8	>50	>50	−	−
S126bF	−	−	>50	−	2.1
K165E	29.2	>50	>50	−	−
R166G	2.8	3.8	5.5	−	−
Y168H	3.3	>50	19.5	−	−
T171A	2.7	>50	>50	−	−
E130D	−	−	−	>50	−
S133L	−	3.4	−	>50	−
K156T	−	3.8	−	>50	−
N159K	−	−	−	>50	−
D77G	−	−	−	−	>50
E119K	−	−	−	−	>50

“−“ indicates less than two fold changes relative to the wild type.

**Table 3:** Data collection and refinement statistics.

	**F55-271**	**65C6 Fab-F55-271**	**AVFLuIgG03 Fab-F55-271**	**100F4 Fab-F55-271**
Data collection*				
Space group	*P*6_1_	*P*22_1_2_1_	*P*6	*P*2_1_2_1_2_1_
				
Cell dimensions				
a, b, c (Å)	93.6, 93.6, 48.4	63.5, 93.0, 134.0	132.7, 132.7, 88.0	88.1, 101.2, 206.3
α, β, γ (°)	90, 90, 120	90, 90, 90	90, 90, 120	90, 90, 90
Resolution (Å)	50-1.50 (1.53–1.50)	50–3.00 (3.07–3.00)	50–3.05 (3.10–3.05)	50–2.80 (2.86–2.80)
*R*_merge_	0.058 (0.321)	0.127 (0.736)	0.194	0.143 (0.624)
*R*_p.i.m._†	0.020 (0.227)	0.057 (0.331)	0.083 (0.730)	0.076 (0.323)
I/σI	32.2 (3.7)	13.9 (3.0)	12.1 (1.3)	10.5 (2.5)
Completeness (%)	98.2 (84.5)	99.8 (100)	100 (100)	99.8 (99.7)
Redundancy	8.4 (2.9)	5.8 (6.0)	7.2 (7.0)	4.3 (4.5)
CC_1/2_	0.857	0.807	0.560	0.750
				
*Refinement*				
Resolution (Å)	30.6–1.50	31.1–3.00	38.9–3.05	40.5–2.82
No. Reflections	38,181	16,304	16,857	45,011
R_work_/R_free_ (%)	16.8/19.0	21.5/26.9	19.9/25.7	20.8/25.7
				
No. atoms				
Protein	1,742	4,894	4,703	9,784
Glycan	28	28		28
Water	290			302
				
B-factors				
Protein	21.3	65.9	63.6	32.5
Glycan	38.3	64.7		27.7
Water	35.5			27.7
				
r.m.s. deviations				
Bond lengths (Å)	0.007	0.011	0.009	0.010
Bond angles (°)	1.108	1.383	1.350	1.385

r.m.s., root mean square.

^*^Numbers in brackets indicate values for the outermost resolution shell.

^†^For *R*_p.i.m._ definition see (Philip Evans 2006, Acta Crystallographica Section D).

## References

[b1] Fauci A. S. Emerging and re-emerging infectious diseases: influenza as a prototype of the host-pathogen balancing act. Cell 124, 665–670 (2006). 16497575 10.1016/j.cell.2006.02.010 PMC7126645

[b2] Park A. W. & Glass K. Dynamic patterns of avian and human influenza in east and southeast Asia. Lancet Infect. Dis. 7, 543–548 (2007). 17646027 10.1016/S1473-3099(07)70186-X

[b3] Salomon R. & Webster R. G. The influenza virus enigma. Cell 136, 402–410 (2009). 19203576 10.1016/j.cell.2009.01.029 PMC2971533

[b4] Xu X., Subbarao K., Cox N. J. & Guo Y. Genetic characterization of the pathogenic influenza A/Goose/Guangdong/1/96 (H5N1) virus: similarity of its hemagglutinin gene to those of H5N1 viruses from the 1997 outbreaks in Hong Kong. Virology 261, 15–19 (1999). 10484749 10.1006/viro.1999.9820

[b5] Li Y. . Continued evolution of H5N1 influenza viruses in wild birds, domestic poultry, and humans in China from 2004 to 2009. J. Virol. 84, 8389–8397 (2010). 20538856 10.1128/JVI.00413-10 PMC2919039

[b6] Smith G. J. . Emergence and predominance of an H5N1 influenza variant in China. Proc. Natl Acad. Sci. USA 103, 16936–16941 (2006). 17075062 10.1073/pnas.0608157103 PMC1636557

[b7] Wan X. F. . Indications that live poultry markets are a major source of human H5N1 influenza virus infection in China. J. Virol. 85, 13432–13438 (2011). 21976646 10.1128/JVI.05266-11 PMC3233185

[b8] Chen H. . Avian flu: H5N1 virus outbreak in migratory waterfowl. Nature 436, 191–192 (2005). 16007072 10.1038/nature03974

[b9] Liu J. . Highly pathogenic H5N1 influenza virus infection in migratory birds. Science 309, 1206 (2005). 16000410 10.1126/science.1115273

[b10] Claas E. C. . Human influenza A H5N1 virus related to a highly pathogenic avian influenza virus. Lancet 351, 472–477 (1998). 9482438 10.1016/S0140-6736(97)11212-0

[b11] Cowling B. J. . Comparative epidemiology of human infections with avian influenza A H7N9 and H5N1 viruses in China: a population-based study of laboratory-confirmed cases. Lancet 382, 129–137 (2013). 23803488 10.1016/S0140-6736(13)61171-X PMC3777567

[b12] Yu H. . Clinical characteristics of 26 human cases of highly pathogenic avian influenza A (H5N1) virus infection in China. PLoS ONE 3, e2985 (2008). 18716658 10.1371/journal.pone.0002985 PMC2515635

[b13] Russell C. J. & Webster R. G. The genesis of a pandemic influenza virus. Cell 123, 368–371 (2005). 16269328 10.1016/j.cell.2005.10.019

[b14] Wang H. . Probable limited person-to-person transmission of highly pathogenic avian influenza A (H5N1) virus in China. Lancet 371, 1427–1434 (2008). 18400288 10.1016/S0140-6736(08)60493-6

[b15] Herfst S. . Airborne transmission of influenza A/H5N1 virus between ferrets. Science 336, 1534–1541 (2012). 22723413 10.1126/science.1213362 PMC4810786

[b16] Imai M. . Experimental adaptation of an influenza H5 HA confers respiratory droplet transmission to a reassortant H5 HA/H1N1 virus in ferrets. Nature 486, 420–428 (2012). 22722205 10.1038/nature10831 PMC3388103

[b17] Zhang Y. . H5N1 hybrid viruses bearing 2009/H1N1 virus genes transmit in guinea pigs by respiratory droplet. Science 340, 1459–1463 (2013). 23641061 10.1126/science.1229455

[b18] Group., W.O.F.H.N.E.W. Continued evolution of highly pathogenic avian influenza A (H5N1): updated nomenclature. Influenza Other Respir. Viruses 6, 1–5 (2011). 22035148 10.1111/j.1750-2659.2011.00298.x PMC5074649

[b19] Li K. S. . Genesis of a highly pathogenic and potentially pandemic H5N1 influenza virus in eastern Asia. Nature 430, 209–213 (2004). 15241415 10.1038/nature02746

[b20] Nicholls J. M. . Tropism of avian influenza A (H5N1) in the upper and lower respiratory tract. Nat. Med. 13, 147–149 (2007). 17206149 10.1038/nm1529

[b21] Caton A. J., Brownlee G. G., Yewdell J. W. & Gerhard W. The antigenic structure of the influenza virus A/PR/8/34 hemagglutinin (H1 subtype). Cell 31, 417–427 (1982). 6186384 10.1016/0092-8674(82)90135-0

[b22] Gerhard W., Yewdell J., Frankel M. E. & Webster R. Antigenic structure of influenza virus haemagglutinin defined by hybridoma antibodies. Nature 290, 713–717 (1981). 6163993 10.1038/290713a0

[b23] Wiley D. C., Wilson I. A. & Skehel J. J. Structural identification of the antibody-binding sites of Hong Kong influenza haemagglutinin and their involvement in antigenic variation. Nature 289, 373–378 (1981). 6162101 10.1038/289373a0

[b24] Wilson I. A., Skehel J. J. & Wiley D. C. Structure of the haemagglutinin membrane glycoprotein of influenza virus at 3A resolution. Nature 289, 366–373 (1981). 7464906 10.1038/289366a0

[b25] Chiu C., Ellebedy A. H., Wrammert J. & Ahmed R. B Cell Responses to Influenza Infection and Vaccination. Curr. Top Microbiol. Immunol. 386, 381–398 (2014). 25193634 10.1007/82_2014_425

[b26] Li C. K., Rappuoli R. & Xu X. N. Correlates of protection against influenza infection in humans–on the path to a universal vaccine? Curr. Opin. Immunol. 25, 470–476 (2013). 23948572 10.1016/j.coi.2013.07.005

[b27] Pica N. & Palese P. Toward a universal influenza virus vaccine: prospects and challenges. Annu. Rev. Med. 64, 189–202 (2013). 23327522 10.1146/annurev-med-120611-145115

[b28] Lingwood D. . Structural and genetic basis for development of broadly neutralizing influenza antibodies. Nature 489, 566–570 (2012). 22932267 10.1038/nature11371 PMC7095019

[b29] Li Y. . Immune history shapes specificity of pandemic H1N1 influenza antibody responses. J. Exp. Med. 210, 1493–1500 (2013). 23857983 10.1084/jem.20130212 PMC3727314

[b30] Wrammert J. . Broadly cross-reactive antibodies dominate the human B cell response against 2009 pandemic H1N1 influenza virus infection. J. Exp. Med. 208, 181–193 (2011). 21220454 10.1084/jem.20101352 PMC3023136

[b31] Wrammert J. . Rapid cloning of high-affinity human monoclonal antibodies against influenza virus. Nature 453, 667–671 (2008). 18449194 10.1038/nature06890 PMC2515609

[b32] Corti D. . A neutralizing antibody selected from plasma cells that binds to group 1 and group 2 influenza A hemagglutinins. Science 333, 850–856 (2011). 21798894 10.1126/science.1205669

[b33] Ekiert D. C. . Antibody recognition of a highly conserved influenza virus epitope. Science 324, 246–251 (2009). 19251591 10.1126/science.1171491 PMC2758658

[b34] Ekiert D. C. . A highly conserved neutralizing epitope on group 2 influenza A viruses. Science 333, 843–850 (2011). 21737702 10.1126/science.1204839 PMC3210727

[b35] Sui J. . Structural and functional bases for broad-spectrum neutralization of avian and human influenza A viruses. Nat. Struct. Mol. Biol. 16, 265–273 (2009). 19234466 10.1038/nsmb.1566 PMC2692245

[b36] Dreyfus C. . Highly conserved protective epitopes on influenza B viruses. Science 337, 1343–1348 (2012). 22878502 10.1126/science.1222908 PMC3538841

[b37] Hu H. . A human antibody recognizing a conserved epitope of H5 hemagglutinin broadly neutralizes highly pathogenic avian influenza H5N1 viruses. J. Virol. 86, 2978–2989 (2012). 22238297 10.1128/JVI.06665-11 PMC3302345

[b38] Sun L. . Generation, characterization and epitope mapping of two neutralizing and protective human recombinant antibodies against influenza A H5N1 viruses. PLoS ONE 4, e5476 (2009). 19421326 10.1371/journal.pone.0005476 PMC2674214

[b39] Zhou B. P., Zhong N. S. & Guan Y. Treatment with convalescent plasma for influenza A (H5N1) infection. N. Engl. J. Med. 357, 1450–1451 (2007). 17914053 10.1056/NEJMc070359

[b40] Zuo T. . Comprehensive analysis of pathogen-specific antibody response in vivo based on an antigen library displayed on surface of yeast. J. Biol. Chem. 286, 33511–33519 (2011). 21795672 10.1074/jbc.M111.270553 PMC3190923

[b41] Shore D. A. . Structural and Antigenic Variation among Diverse Clade 2 H5N1 Viruses. PLoS ONE 8, e75209 (2013). 24086467 10.1371/journal.pone.0075209 PMC3785507

[b42] Qian M. . Unraveling of a neutralization mechanism by two human antibodies against conserved epitopes in the globular head of H5 hemagglutinin. J. Virol. 87, 3571–3577 (2013). 23269809 10.1128/JVI.01292-12 PMC3592130

[b43] Zhu X. . A unique and conserved neutralization epitope in H5N1 influenza viruses identified by an antibody against the A/Goose/Guangdong/1/96 hemagglutinin. J. Virol. 87, 12619–12635 (2013). 24049169 10.1128/JVI.01577-13 PMC3838140

[b44] Wang T. T. . Vaccination with a synthetic peptide from the influenza virus hemagglutinin provides protection against distinct viral subtypes. Proc. Natl Acad. Sci. USA 107, 18979–18984 (2010). 20956293 10.1073/pnas.1013387107 PMC2973924

[b45] Wang G. . DNA prime and virus-like particle boost from a single H5N1 strain elicits broadly neutralizing antibody responses against head region of H5 hemagglutinin. J. Infect. Dis. 209, 676–685 (2014). 23911711 10.1093/infdis/jit414

[b46] Chiu C. . Cross-reactive humoral responses to influenza and their implications for a universal vaccine. Ann. NY Acad. Sci. 1283, 13–21 (2013). 23405860 10.1111/nyas.12012

[b47] Xu R. . Structural basis of preexisting immunity to the 2009 H1N1 pandemic influenza virus. Science 328, 357–360 (2010). 20339031 10.1126/science.1186430 PMC2897825

[b48] Ekiert D. C. . Cross-neutralization of influenza A viruses mediated by a single antibody loop. Nature 489, 526–532 (2012). 22982990 10.1038/nature11414 PMC3538848

[b49] Whittle J. R. . Broadly neutralizing human antibody that recognizes the receptor-binding pocket of influenza virus hemagglutinin. Proc. Natl Acad. Sci. USA 108, 14216–14221 (2011). 21825125 10.1073/pnas.1111497108 PMC3161572

[b50] Lee P. S. . Heterosubtypic antibody recognition of the influenza virus hemagglutinin receptor binding site enhanced by avidity. Proc. Natl Acad. Sci. USA 109, 17040–17045 (2012). 23027945 10.1073/pnas.1212371109 PMC3479480

[b51] DiazGranados C. A. . Efficacy of high-dose versus standard-dose influenza vaccine in older adults. N. Engl. J. Med. 371, 635–645 (2014). 25119609 10.1056/NEJMoa1315727

[b52] Fiore A. E., Bridges C. B. & Cox N. J. Seasonal influenza vaccines. Curr. Top Microbiol. Immunol. 333, 43–82 (2009). 19768400 10.1007/978-3-540-92165-3_3

[b53] Khurana S. . Vaccines with MF59 adjuvant expand the antibody repertoire to target protective sites of pandemic avian H5N1 influenza virus. Sci. Transl. Med. 2, 15ra15 (2010). 10.1126/scitranslmed.3000624 20371470

[b54] Khurana S. . MF59 adjuvant enhances diversity and affinity of antibody-mediated immune response to pandemic influenza vaccines. Sci. Transl. Med. 3, 85ra48 (2011). 10.1126/scitranslmed.3002336 PMC3501657 21632986

[b55] Khurana S. . Vaccine-induced anti-HA2 antibodies promote virus fusion and enhance influenza virus respiratory disease. Sci. Transl. Med. 5, 200ra114 (2013). 10.1126/scitranslmed.3006366 23986398

[b56] Song L. . Superior efficacy of a recombinant flagellin:H5N1 HA globular head vaccine is determined by the placement of the globular head within flagellin. Vaccine 27, 5875–5884 (2009). 19654064 10.1016/j.vaccine.2009.07.060 PMC3571653

[b57] Verma S. . Oligomeric recombinant H5 HA1 vaccine produced in bacteria protects ferrets from homologous and heterologous wild-type H5N1 influenza challenge and controls viral loads better than subunit H5N1 vaccine by eliciting high-affinity antibodies. J. Virol. 86, 12283–12293 (2012). 22951833 10.1128/JVI.01596-12 PMC3486503

[b58] Xuan C. . Structural vaccinology: structure-based design of influenza A virus hemagglutinin subtype-specific subunit vaccines. Protein Cell 2, 997–1005 (2011). 22231357 10.1007/s13238-011-1134-y PMC4875251

[b59] Otwinowski Z. & Minor W. Processing of X-ray diffraction data collected in oscillation mode. Macromol. Crystallogr. A 276, 307–326 (1997). 10.1016/S0076-6879(97)76066-X 27754618

[b60] McCoy A. J. Solving structures of protein complexes by molecular replacement with Phaser. Acta Crystallogr. Sect. D-Biol. Crystallogr. 63, 32–41 (2007). 17164524 10.1107/S0907444906045975 PMC2483468

[b61] Adams P. D. . PHENIX: building new software for automated crystallographic structure determination. Acta Crystallogr. Sect. D-Biol. Crystallogr. 58, 1948–1954 (2002). 12393927 10.1107/s0907444902016657

[b62] Laskowski R. A., Macarthur M. W., Moss D. S. & Thornton J. M. Procheck - a Program to Check the Stereochemical Quality of Protein Structures. J. Appl. Crystallogr. 26, 283–291 (1993).

[b63] Delano W. L. Pymol Molecular Graphics System. http://pymol.org/ (2002).

