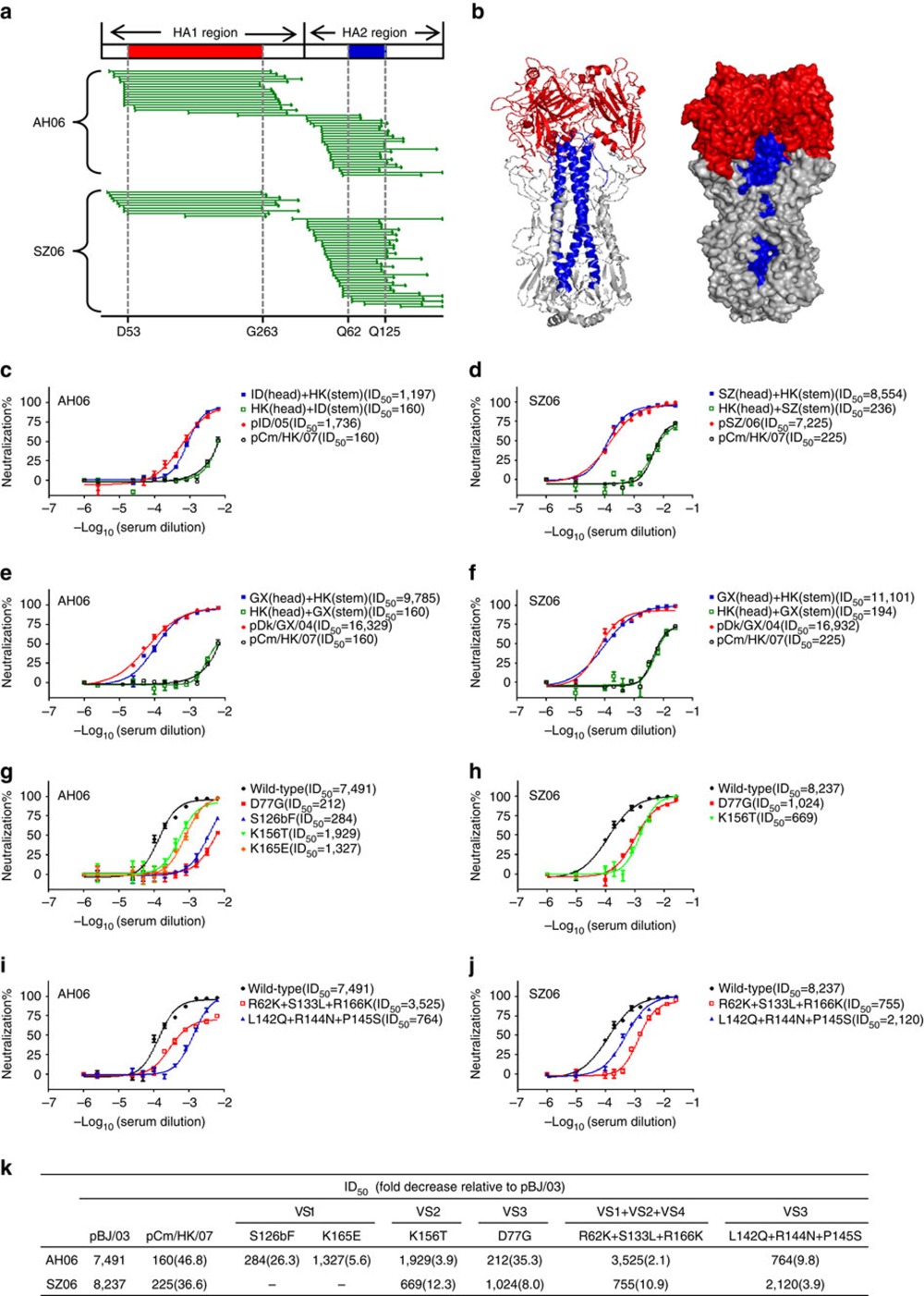# Erratum: Comprehensive analysis of antibody recognition in convalescent humans from highly pathogenic avian influenza H5N1 infection

**DOI:** 10.1038/ncomms10757

**Published:** 2016-02-15

**Authors:** Teng Zuo, Jianfeng Sun, Guiqin Wang, Liwei Jiang, Yanan Zuo, Danyang Li, Xuanling Shi, Xi Liu, Shilong Fan, Huanhuan Ren, Hongxing Hu, Lina Sun, Boping Zhou, Mifang Liang, Paul Zhou, Xinquan Wang, Linqi Zhang

*Nature Communications* 6: Article number: 885510.1038/ncomms9855 (2015); Published 12042015; Updated 02152016

In Fig. 4k of this Article, the first row in the far-right column of the table contains a typographical error and should read ‘VS3'. The correct version of Fig. 4 appears below.

**Figure 4**