# Supplementary material for: Comprehensive analysis of antibody recognition in convalescent humans from highly pathogenic avian influenza H5N1 infection
Source: Nat Commun. 2015 Dec 4;6:8855. doi: 10.1038/ncomms9855 (PMC4686829; doi:10.1038/ncomms9855)
Supplement: Supplementary Information — Supplementary Figures 1-9, Supplementary Tables 1-8 and Supplementary References [file ncomms9855-s1.pdf]

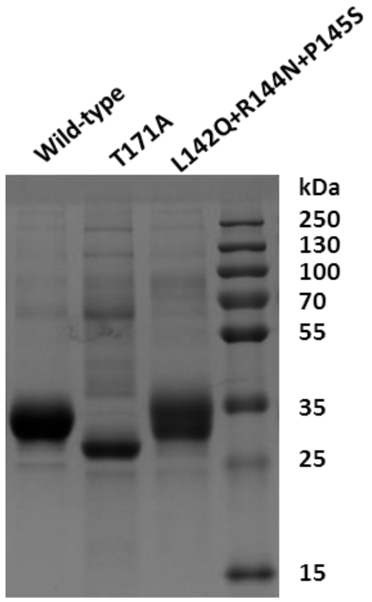

Supplementary Figure 1. The effects of mutations on N-linked glycosylation of HA. The wild type and mutated (T171A and L142Q+R144N+P145S) globular head of A/Beijing/01/03 HA were produced in 293T cells, purified and analyzed by reducing SDS-PAGE.

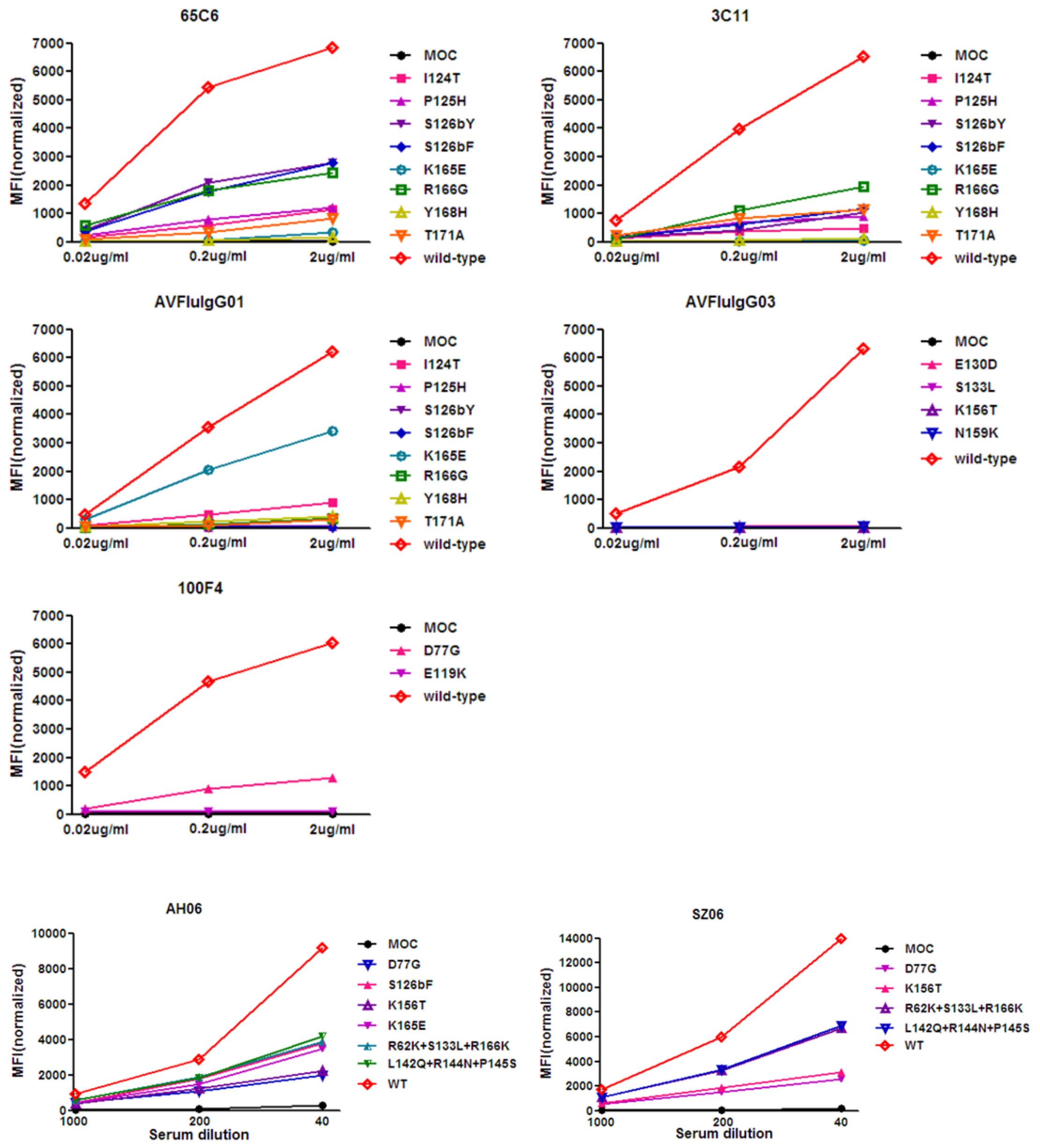

Supplementary Figure 2. Impact of mutations on the binding activities of the five mAbs and two convalescent serum samples. 293T cells were transfected with wild type or mutated HA expression vector followed by staining with a series dilutions of the 5 mAbs (65C6, 3C11, AVFluIgG01, AVFluIgG03, and 100F4) or convalescent serum samples (AH06 and SZ06). The binding activity was measured by mean fluorescence intensity (MFI) through FACS analysis. MFI was normalized by a stem-specific mAb F10.

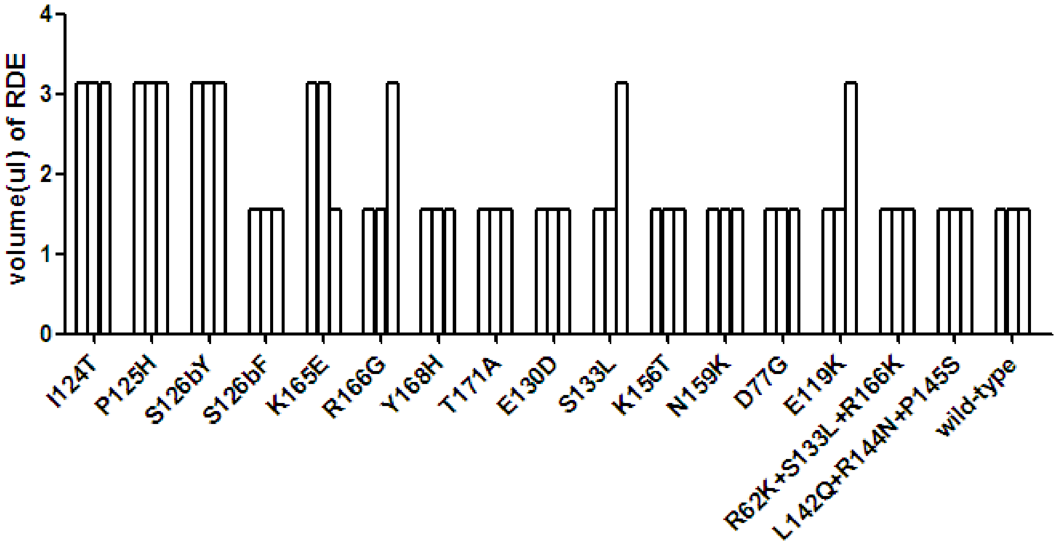

Supplementary Figure 3. The effects of mutations on receptor binding avidity. Cellular receptor binding avidity of wild type and mutated HAs was determined by hemagglutination of SPF chicken erythrocytes pre-treated with receptor-destroying enzyme (RDE). Data represents the maximal amount of RDE that allowed full agglutination. All tests were done in triplicates. As the maximal amounts of RDE that allowed full agglutination among wild type and mutants were within 2-fold difference, we concluded that introduced HA mutations did not significantly alter receptor binding avidity.

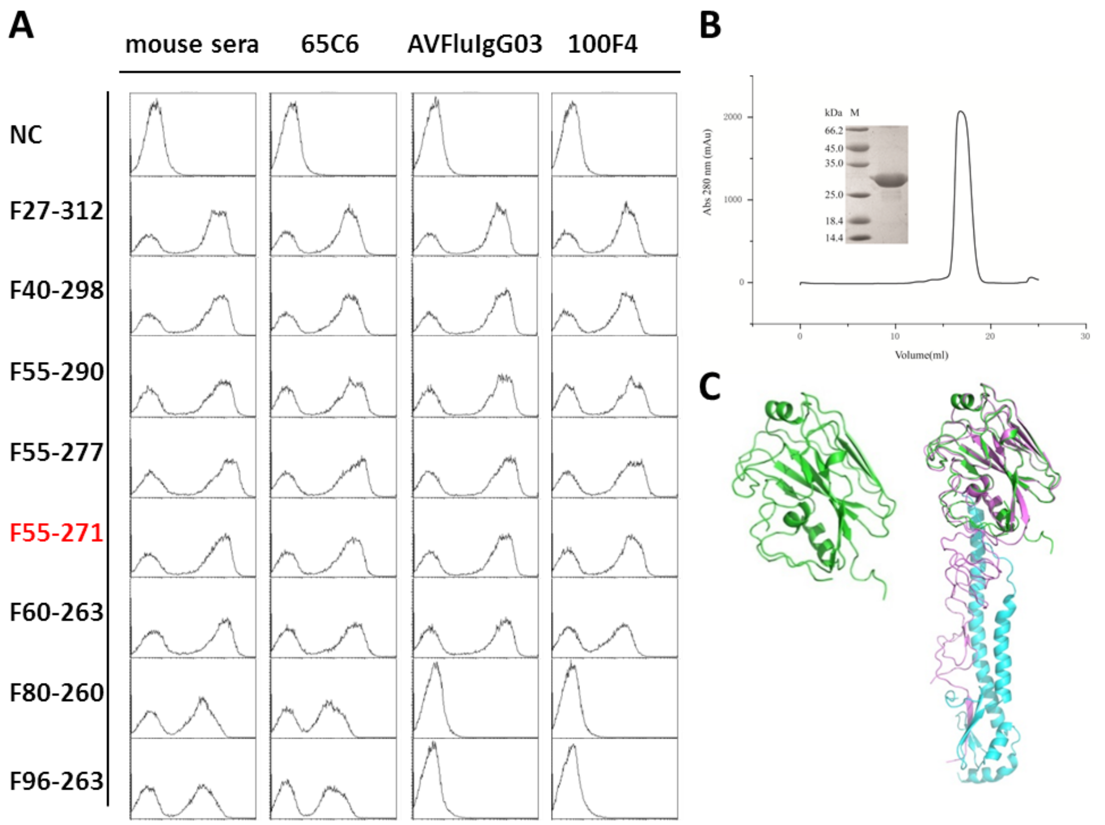

Supplementary Figure 4. Selection, expression and crystal structure of globular head derived from A/Anhui/1/05 HA. (A) Binding activities of 8 different HA fragments to 65C6, AVFluIgG03 and 100F4 analyzed by FACS. Mouse immune sera with H5N1 HA were used as a positive control. F55-271 labeled in red was selected to express globular head in insect cells due to its relative shorter length while maintaining strong binding to the 3 mAbs. **(B)** F55-271 was purified by size exclusion chromatography on a Superdex-200 column and analyzed by reducing SDS-PAGE. **(C)** The crystal structure of F55-271 and the superimposition thereof onto the monomeric A/Anhui/1/05 HA ectodomain. NC: negative control yeast clone expressing an irrelevant antigen.

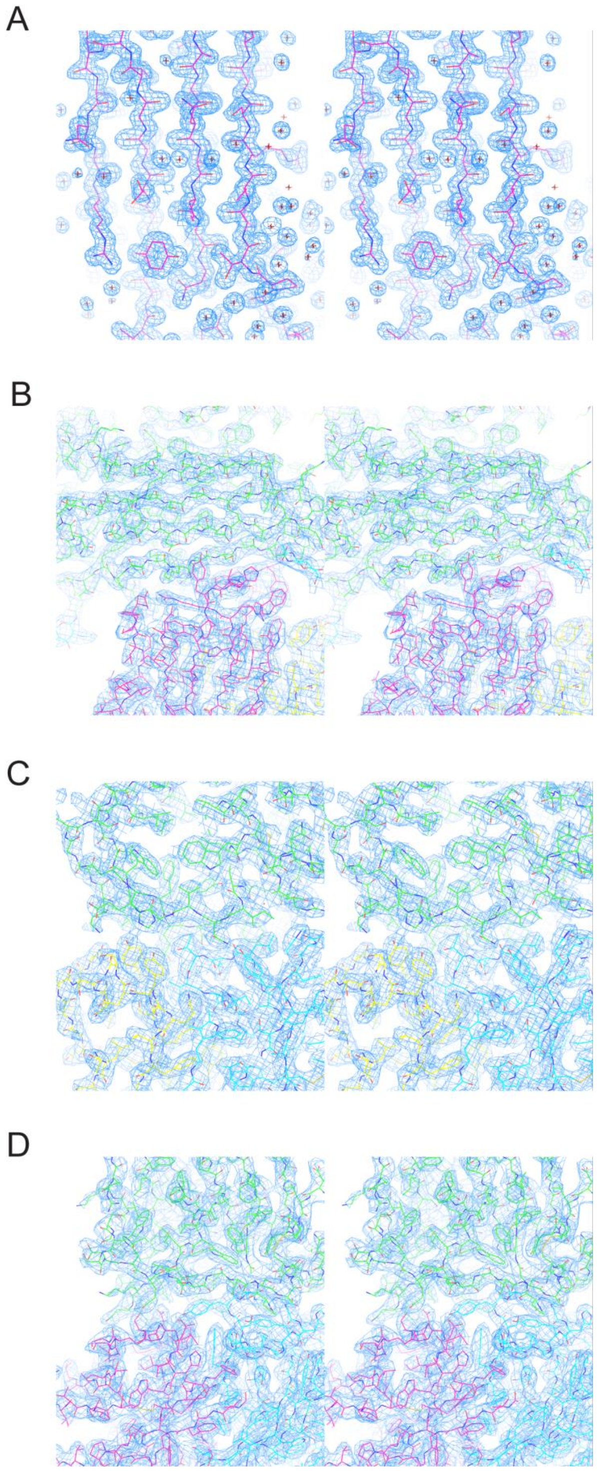

Supplementary Figure 5. 2Fo-Fc electron densities contoured at 1.2 σ of H5 HA globular head structure (**A**) and its complexes with the Fab of 65C6 (**B**), 100F4 (**C**) and AVFluIgG03 **(D).** In the last three panels, the H5 HA globular head is presented as lines in green color. The heavy and light chains of antibody are also presented as lines in other two different colors.

.

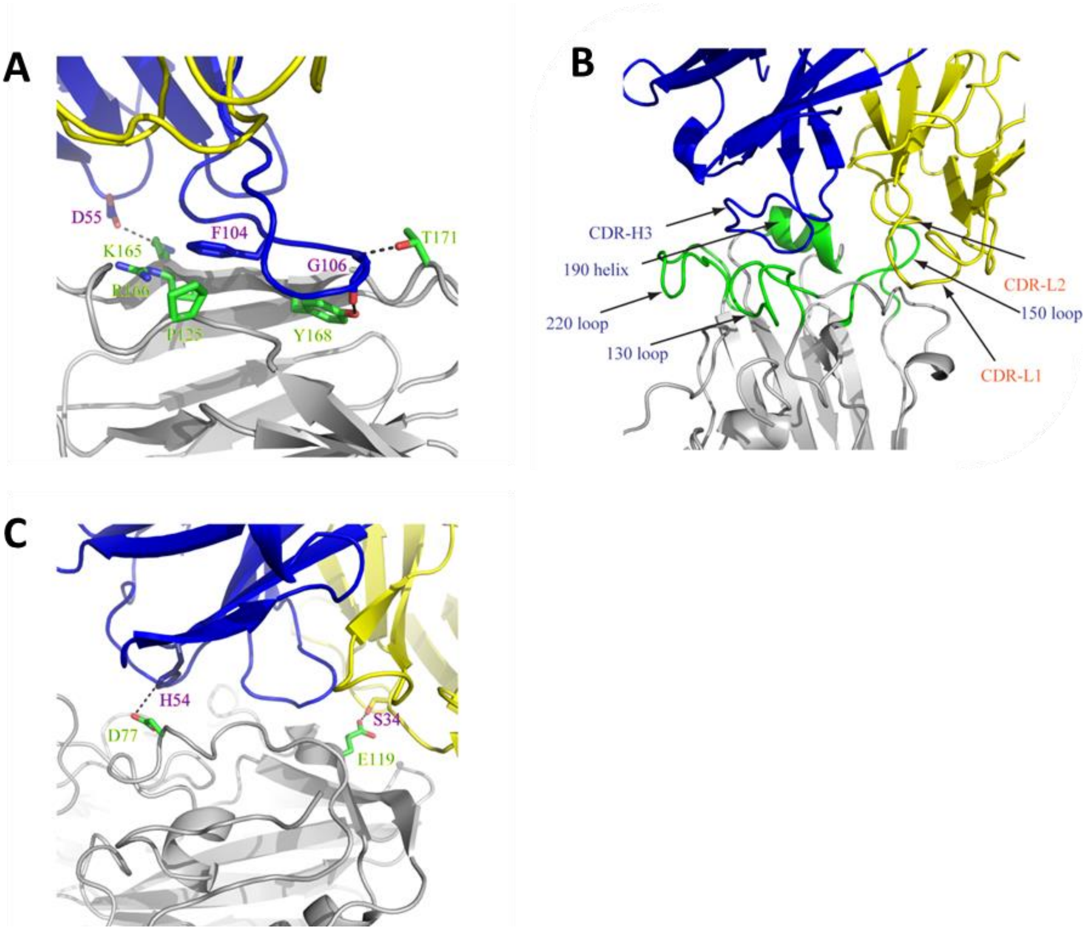

Supplementary Figure 6. Closer view of the interactions between HA globular head and each of the three mAbs. In all three panels, the globular head is colored in grey, while the heavy and light chains of the mAbs in blue and yellow, respectively. **(A)** The long CDR-H3 of 65C6 stretched parallel with β strand 162-171 and loop 121-129 of the globular head. F104 of CDR-H3 formed hydrophobic interactions with P125 and R166. G106 of CDR-H3 formed hydrogen bonds with Y168 and T171. D55 of 65C6 CDR-H2 interacted with K165 through a salt-bridge. **(B)** The 130 loop, 150 loop, 190 helix, and 220 loop surrounding the RBS of the globular head are shown in green. The long CDR-H3 of AVFluIgG03 interacted with the 130 loop, 150 loop, 190 helix, and 220 loop. The CDR-L1 and CDR-L2 interacted with the 130 and 150 loops, respectively. **(C)** Hydrogen bonds was formed between D77 of globular head and H54 of 100F4 CDR-H2, and between E119 of globular head and S34 of 100F4 CDR-L1.

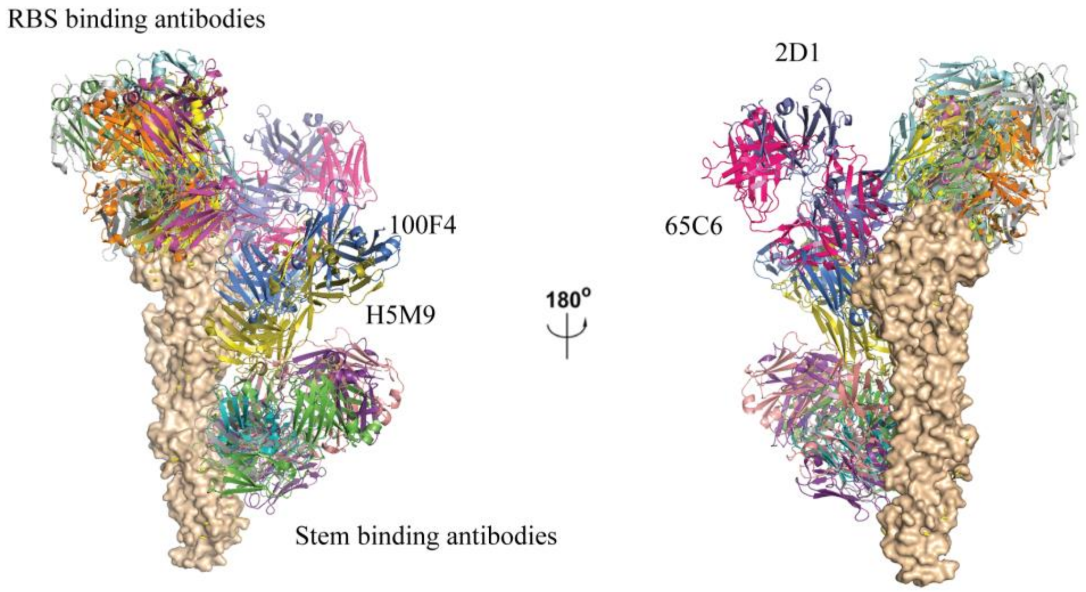

Supplementary Figure 7. Five major modes of recognition by neutralizing mAbs against HA. A total of 16 solved HA and antibody complex structures were docked onto monomeric A/Anhui/1/05 HA (showed in surface and colored in wheat). Seven antibodies including C05 (PDB 4FP8), F045-092 (PDB 4O5I), S139/1 (PDB 4GMS), 1F1 (PDB 4GXU), 5J8 (PDB 4M5Z), CH65 (PDB 3SM5) and AVFluIgG03 bind to the RBS of HA. Antibodies 2D1 (PDB 3LZF) and 65C6 recognized the opposite side of RBD whereas 100F4 and H5M9 (PDB 4MHH) bind to regions further away from the RBS. Five antibodies including Fab 39.29 (PDB 4KVN), unnamed (PDB 4UBD), CR6261 (PDB 3GBM), F10 (PDB 3FKU) and FI6v3 (PDB 3ZTJ) converged to a conserved region in the stem of HA.

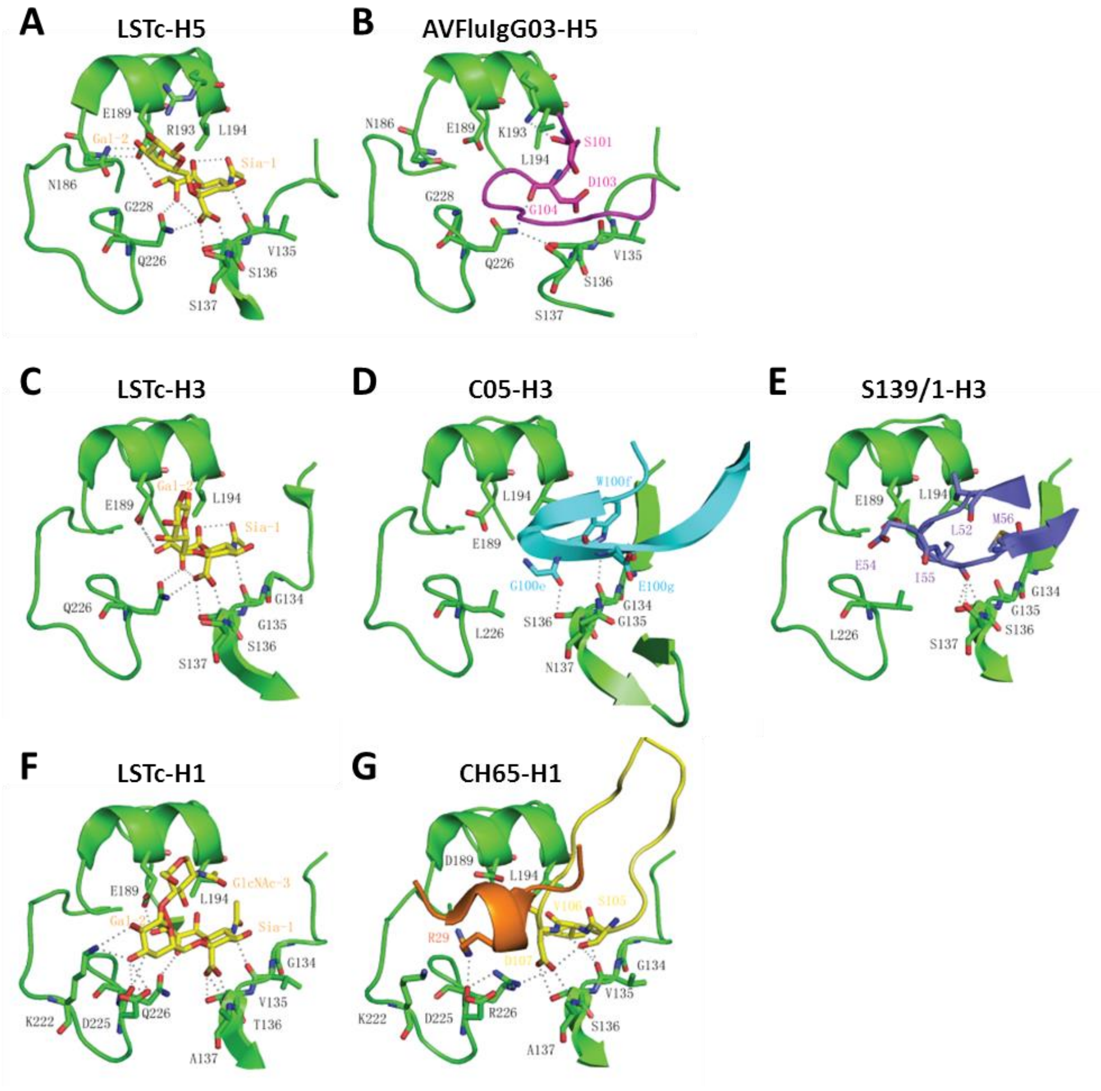

Supplementary Figure 8. Comparison of RBS-targeting mAbs and receptor mimicry. Schematic representation of LSTc (colored in yellow) bound into the RBS of **(A)** H5 A/Indonesia/5/2005 (PDB 4K64), **(C)** H3 A/Duck/Ukraine/1/63 (PDB 1MNQ), and **(F)** H1 A/Puerto Rico/8/1934 (PDB 1RVZ). **(B)** The interactions of AVFluIgG03 CDR-H3 (colored in purple) within the RBS of A/Anhui/1/05 HA. **(D)** The interactions of C05 CDR-H3 (colored in cyans) within the RBS of A/Hong Kong/1/1968 HA (PDB 4FP8). **(E)** The interactions of S139/1 CDR-H2 (colored in purpleblue) within the RBS of A/Victoria/3/1975 HA (PDB 4GMS). (G) The interactions of CH65 CDR-L1 (colored in orange) and CDR-H3 (colored in yellow) within the RBS of A/Solomon Islands/3/2006 HA (PDB 3SM5). In all panels, the RBS is colored in green and dashed lines represent polar interactions between LSTc and RBS or mAbs with RBS.

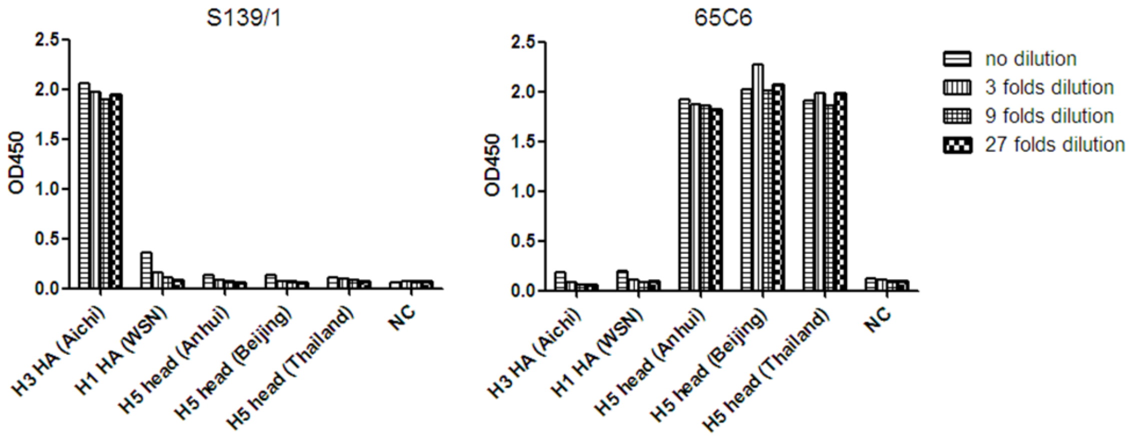

Supplementary Figure 9. Binding activities of mAb S139/1 against various HA subtypes by ELISA.

Supplementary Table 1. **Spatial segregation of mutant residues into buried or exposed relative to the HA surface**

Identified residue mutations									
65C6		3C11		AVFluIgG01		AVFluIgG03		100F4	
Buried	Exposed	Buried	Exposed	Buried	Exposed	Buried	Exposed	Buried	Exposed
L154H	I123T	C67R	K120E	L209P	K120E	L71H/P	H129bR	L70H	D77G
N170D	I124S	F95Y	I123T	G249E	I124T	G72R	E130D	L71F	E119K
W180R	P125H	L101P	I124T	P254S	P125H	C97S/R	S133L	I87T	Y141C
G181E	S126bY	L172P	W127R	Y257C	S126bF/P	P99L	V135E	E89G	F147L
I182T	V151E	L173S	W153R		W127R	L108Q	S146P	W153R	E255G
Y201C	K156T	W176L	K156E/T		H129bR	C139R	F147L/S	R229G	Y257C
S203P	I164T	Y191F/H	K157E		W153R	L150P	F148S	F232L	K258E
F232L	K165E	F228S	I164T		K157E	H184R	N150D	I252M	
W234C	R166G	W230C	K165R		I164T	Y195C/N	K156E		
N250S	S167G	I239T	Y168H/C		R166S/G	F232L	N159K		
	Y168H	G245E	H183Q		Y168H	N250D	H183R		
	T171A	N246S	Q191R		T171A		D187E		
	Q191R	I248F	F245Y/L				Q191L		
		P250S					L194H		

Supplementary Table 2. Contacting residues at the binding interface (d ⩽4.0 Å) between the globular head and the 3 mAbs

F55-271	65C6		F55-271	AVFluIgG03		F55-271	100F4	
	H chain	L chain		H chain	L chain		H chain	L chain
I121	G106		E130		N53	D77	H54	
Q122	S107				S54		S56	
	G108		S132		G32		H58	
I123	F104				D34	E78	H58	
	G108		G134	D103		I80	Y35	
P125	F104		V135	D103			H54	
	G108			Y108			L102	
S126b	F104		S136	D103		N81	Y35	
S128		W94	S137	G106			L102	
D129	Y57			Y108			T103	
	N59		T144	L110			T104	
P162	Y57		P145	Y108		H117		Y33
T163	R101			L110				Y93
K165	Y31		W153	D103		E119	T104	G32
	D55		I155	Y102			V105	Y33
	R101		K156		Y51			S34
	G102				N55	K120	L102	
R166	G102		K157		S54		T103	
	F104		N158		S54		T104	
S167	G102		N159		S54	I121		S34
	H103				N55	Q122	L101	Y51
	F104				R56		T103	
Y168	F104		E190	G104		K126	S33	
	Y105		K193	S101	Y51	Y141	S30	
	G106			Y102			S31	
N169	H103		L194	Y102			G55	
	F104		K222	G105			S56	
	Y105		G225	G105		Q142	G55	
	G106		Q226	D103		R149	G32	
T171	Y105			G105			H54	
	G106					T171		S53
N172	G106					N172		S53
N244	Y31					Q173		K68
E246	Y31					E174		G32
						Y255a	L101	
							L102	
							T103	
						K258		G32
								S34
								K68
						V260		A31
								G32
								Y33
						K261		A31
								Y33
								S95

Supplementary Table 3. **Residue polymorphism within the epitopes of 65C6, AVFluIgG03 and 100F4 among 1663 influenza H5N1 HAs**

mAbs	Epitope	No. of times an amino acid occurs at indicated position																				conservation
		A	C	D	E	F	G	H	I	K	L	M	N	P	Q	R	S	T	V	W	Y	（%）
65C6	121	0	0	0	0	0	0	0	1656	0	4	1	0	0	0	0	0	1	1	0	0	99.6
	122	0	0	0	1	0	0	1	0	64	2	0	0	1	1524	70	0	0	0	0	0	91.6
	123	0	0	0	0	1	0	0	1661	0	0	0	0	0	0	0	0	0	1	0	0	99.9
	125	0	0	0	0	0	0	1	0	0	0	0	0	1649	0	2	11	0	0	0	0	99.2
	126b	0	1	1	0	10	0	2	0	0	0	0	0	1	0	0	1629	0	0	0	19	98.0
	128	13	0	0	0	2	0	0	0	0	3	0	0	228	0	0	1415	0	1	0	0	85.1
	129	0	0	1143	23	0	4	1	0	0	0	0	273	0	0	1	217	0	0	0	1	68.7
	162	0	0	0	0	0	0	0	0	0	1	0	0	1660	0	0	1	1	0	0	0	99.8
	163	1	0	0	0	0	0	0	27	0	4	0	0	35	0	0	0	1595	0	0	0	95.9
	165	0	0	0	11	0	0	0	0	1642	0	0	3	0	2	3	0	2	0	0	0	98.7
	166	0	0	0	35	0	3	0	90	525	0	11	0	0	0	949	19	1	30	0	0	57.1
	167	0	0	8	0	0	120	0	0	0	0	0	51	0	0	1	1435	47	1	0	0	86.3
	168	0	0	0	0	1	0	0	0	0	0	0	0	0	0	0	0	0	0	0	1661	99.9
	169	0	0	0	0	0	0	94	0	5	0	0	1526	0	0	0	4	33	0	0	1	91.8
	171	15	0	0	0	0	0	0	5	0	0	0	0	0	0	0	0	1643	0	0	0	98.8
	172	0	0	8	0	0	4	0	0	0	0	0	1645	0	0	0	5	1	0	0	0	98.9
	244	0	0	1	0	0	0	164	0	0	0	0	1439	0	0	1	52	2	0	0	3	86.5
	246	0	0	37	1623	0	1	0	0	0	0	0	0	0	1	0	0	0	0	0	0	97.6
AVFluIgG03	130	0	0	103	1554	0	3	0	0	0	0	0	2	0	0	0	0	0	0	0	1	93.4
	132	0	0	0	0	2	0	0	0	0	0	0	0	0	0	0	1653	5	0	0	0	99.4
	133	3	0	0	0	0	1	0	0	0	618	3	0	0	0	0	858	0	1	0	0	51.6
	134	0	0	0	0	0	1660	0	0	0	0	0	0	0	0	1	0	0	0	2	0	99.8
	135	1	0	0	0	0	2	0	0	0	0	2	0	0	0	0	0	0	1658	0	0	99.7
	136	0	0	0	0	0	1	0	0	0	0	0	0	0	0	1	1661	0	0	0	0	99.9
	137	329	0	0	0	0	0	0	0	0	1	0	0	0	0	0	1331	1	0	0	0	80.0
	144	3	0	4	17	0	83	0	4	328	0	21	231	0	23	657	108	162	22	0	0	39.5

	145	2	0	0	0	1	0	1	0	0	6	0	0	485	0	0	1162	6	0	0	0	69.9
	153	0	0	0	0	0	0	0	0	0	0	0	0	0	0	0	0	0	0	1663	0	100.0
	155	0	0	0	0	0	0	0	1434	0	13	0	0	0	0	0	0	205	11	0	0	86.2
	156	0	0	0	5	0	1	0	1	1650	0	0	2	0	2	0	0	2	0	0	0	99.2
	157	0	0	0	0	0	0	0	0	1655	0	0	0	0	0	8	0	0	0	0	0	99.5
	158	0	0	458	1	0	9	0	0	1	0	0	1190	0	0	0	2	0	0	0	0	71.6
	159	9	0	205	0	0	6	1	0	0	0	0	944	0	0	0	497	1	0	0	0	56.8
	193	0	0	10	4	0	7	0	0	660	0	22	10	0	10	937	3	0	0	0	0	56.3
	194	0	0	0	0	0	0	0	97	0	1563	0	0	0	0	0	0	0	3	0	0	94.0
	222	0	0	0	4	0	0	0	0	1645	0	0	0	0	5	8	0	0	0	0	0	98.9
	225	0	0	0	1	0	1658	0	0	0	0	0	0	0	0	3	0	0	0	0	0	99.7
	226	0	0	0	0	0	0	0	0	1	3	0	0	1	1656	1	0	0	0	0	0	99.6
100F4	77	0	0	1657	0	0	5	0	0	0	0	0	0	0	0	0	0	0	0	0	0	99.6
	78	6	0	1	1635	0	0	0	0	20	0	0	0	0	0	0	0	0	0	0	0	98.3
	80	0	0	0	0	4	0	1	902	0	638	0	13	51	0	0	2	49	2	0	0	54.2
	81	0	0	41	0	0	0	2	0	5	0	0	1611	0	0	2	1	0	0	0	0	96.9
	117	0	0	0	0	0	0	1567	0	0	0	0	0	0	0	96	0	0	0	0	0	94.2
	119	0	0	1	1646	0	0	0	0	16	0	0	0	0	0	0	0	0	0	0	0	99.0
	120	0	0	0	1	0	1	0	0	1661	0	0	0	0	0	0	0	0	0	0	0	99.9
	121	0	0	0	0	0	0	0	1656	0	4	1	0	0	0	0	0	1	1	0	0	99.6
	122	0	0	0	1	0	0	1	0	64	2	0	0	1	1524	70	0	0	0	0	0	91.6
	126	119	0	0	0	0	0	0	0	0	1571	0	0	5	1	0	86	0	0	0	0	94.5
	141	0	0	0	0	0	0	8	0	0	0	0	0	0	0	0	1	0	0	0	1654	99.5
	142	0	0	0	0	0	0	44	3	1	247	4	73	0	1288	0	1	0	0	0	1	77.5
	149	0	0	0	0	0	0	0	0	4	0	0	0	0	0	1656	1	0	0	1	0	99.6
	171	15	0	0	0	0	0	0	5	0	0	0	0	0	0	0	0	1643	0	0	0	98.8
	172	0	0	8	0	0	4	0	0	0	0	0	1645	0	0	0	5	1	0	0	0	98.9
	173	0	0	0	0	0	1	2	0	40	0	3	0	5	1532	42	0	0	38	0	0	92.1
	174	1	0	1	1654	0	1	0	0	6	0	0	0	0	0	0	0	0	0	0	0	99.5

255a	0	0	0	0	0	0	2	0	1	0	0	576	0	0	0	0	0	0	0	1083	65.1
258	0	0	0	1	0	0	0	9	1649	0	0	0	0	3	0	0	0	0	0	0	99.2
260	2	1	0	0	0	0	0	8	0	0	0	0	0	0	0	0	0	1651	0	0	99.3
261	0	0	0	0	0	0	0	0	1658	0	0	0	0	2	2	0	0	0	0	0	99.7

Supplementary Table 4. **Immunogen, name, origin, epitopes and mapping methods of reported neutralizing mAbs against H5N1**

Immunogen source	Names of nmAbs	Source	Epitope mapping methods	Epitope residue position	Reference
A/Viet Nam/CL26/2004(H5N1,clade 1)	FLA5.10, FLD21.140	Human	Random peptide library	122-125,126b-128, 130-133, 168-171	1
A/Viet Nam/CL115/2004(H5N1, clade 1)					
A/Turkey/Ontario/7732/66(H5N9)	77B1, 56GS, 42C3 etc.	Mouse	Escape mutant	46, 62, 122, 145, 156, 186, 193	2
A/chicken/Pennsylvania/1370/83(H5N2)	cp58,176/26, cp55 etc.	Mouse	Escape mutant	57, 129, 130, 133, 140, 142, 144, 145, 156, 157, 189	3
A/chicken/Pennsylvania/8125/83(H5N2)					
A/Vietnam/1203/04(H5N1, clade 1)	VN04-(2, 8, 9) etc.	Mouse	Escape mutant	126b, 143-145, 155, 156, 160, 166, 187, 193	4
A/chicken/Hong Kong/YU22/2002(H5N1, clade 8)	20A11, 16F13, 13D4 etc.	Mouse	Site-directed mutagenesis	93, 129, 133, 187, 193, 216, 266, 280	5
A/duck/ Viet Nam /S654/2005(H5N1, clade 1)					
A/duck/Indonesia/MS/2004(H5N1, clade 2.1)					
A/bar headded goose/Qinghai/15C/2005(H5N1, clade 2.2)					
A/Viet Nam/568/2005(H5N1, clade 2.3)					
A/goose/Guangdong/1/96(H5N1, clade 0)	H5M6, H5M7, H5M8 etc.	Mouse	Site-directed mutagenesis	91, 129, 130, 142, 266, 272	6
A/chicken/Hong Kong/YU22/2002(H5N1, clade 8)	13D4	Mouse	Escape mutant	156, 186, 193	7
A/duck/ Viet Nam /S654/2005(H5N1, clade 1)					
A/duck/Indonesia/MS/2004(H5N1, clade 2.1)					
A/bar headded goose/Qinghai/15C/2005(H5N1, clade 2.2)					
A/Viet Nam/568/2005(H5N1, clade 2.3)					
A/Indonesia/CDC669/06(H5N1, clade 2.1)	2D9	Mouse	Escape mutant	193, 227	8
A/Aichi/2/68(H3N2)	S139/1	Mouse	Escape mutant	156, 158, 193	9
A/Indonesia/TLL014/2006(H5N1, clade 2.1)	3B1	Mouse	Escape mutant	159, 193	10
A/duck/Novosibirsk/56/05(H5N1, clade 2.2)	4F11, 7E11, 6F3 etc.	Mouse	Escape mutant	120, 122, 124, 125, 126a, 126b, 128, 143, 145, 166	11
A/Indonesia/CDC669/2006(H5N1, clade 2.1)	3H11, 4C2, 2D9 etc.	Mouse	Escape mutant	142-144, 159, 193, 227	12
A/Indonesia/TLL014/2006(H5N1, clade 2.1)					
A/Vietnam/1203/2004(H5N1, clade 1)	NR2728	Mouse	Random mutagenesis library	126b, 129a, 161, 164, 166	13
A/Viet Nam/1194/2004(H5N1, clade 1)	mAb12-1G6	Mouse	Escape mutant	144-149	14
A/Anhui/1/2005(H5N1, clade 2.3.4)	HA-7	Mouse	Cryo-EM	81-83, 117-122	15

Supplementary Table 5. Residue polymorphism within the VS1 among different clades and subclades of H5N1

Virus strain	Clade	Mean ID_{50}	Residue at VS1																							
			122	123	124	125	126	126a	126b	127	128	129	129a	162	163	164	165	166	167	168	169	170	171	172	173	174
A/duck/Guangxi/1378/2004	5	16631	Q	I	I	P	K	S	S	W	S	N	H	P	T	I	K	R	S	Y	N	N	T	N	Q	E
A/chicken/Henan/16/2004	8	14414	-	-	-	-	-	-	-	-	-	-	-	-	-	-	-	-	-	-	-	-	-	-	-	-
A/goose/Shantou/1621/2005	9	10724	-	-	-	-	-	-	-	-	-	-	-	-	-	-	-	-	-	-	-	-	-	-	-	-
A/silky chicken/Hong Kong/SF189/2001	3	10597	-	-	-	-	-	-	-	-	-	-	-	-	-	-	-	-	-	-	-	-	-	-	-	-
A/Beijing/01/2003	7	7864	-	-	-	-	-	-	-	-	-	-	-	-	-	-	-	-	-	-	-	-	-	-	-	-
A/blackbird/Hunan/1/2004	6	7256	-	-	-	-	-	-	-	-	-	D	-	-	-	-	-	-	-	-	-	-	-	-	-	A
A/Turkey/65596/2006	2.2.1	5580	-	-	-	-	-	-	-	-	-	D	-	-	-	-	-	-	-	-	-	-	-	-	-	-
A/Indonesia/5/2005	2.1.3.2	5175	-	-	-	-	-	-	-	-	-	D	-	-	-	-	-	K	-	-	-	-	-	-	-	-
A/Shenzhen/406H/2006	2.3.4	4953	-	-	-	-	-	-	-	-	-	D	-	-	-	-	-	-	-	-	-	-	-	-	-	-
A/chicken/Korea/es/2003	2.5	4640	-	-	-	-	-	-	-	-	-	D	-	-	-	-	-	-	-	-	-	-	-	-	-	-
A/Thailand/(KAN-1)/2004	1	4094	-	-	-	-	-	-	-	-	-	S	-	-	-	-	-	-	-	-	-	-	-	-	-	-
A/chicken/Guangxi/12/2004	2.4	2578	-	-	-	-	-	-	-	-	-	D	-	-	-	-	-	I	-	-	-	-	-	-	-	-
A/Chicken/Vietnam /NCVD-16/08	7.1	1986	K	-	-	-	-	-	Y	-	-	-	-	-	P	-	-	V	N	-	T	-	-	-	-	-
A/Hong Kong/156/1997	0	1933	-	-	-	-	-	-	-	-	-	-	-	-	-	-	-	-	-	-	-	-	-	-	-	-
A/Chicken/Vietnam/NCVD-03/08	7.1	1814	K	-	-	-	-	-	Y	-	-	-	-	-	P	-	-	V	N	-	T	-	-	-	-	-
A/goose/Guiyang/337/2006	4	609	-	-	-	-	-	-	-	-	P	-	-	-	-	-	-	-	-	-	-	-	-	-	-	-
A/common magpie/Hong Kong/5052/2007	2.3.2.1	192	-	-	-	-	-	D	-	-	-	D	-	-	-	-	-	K	-	-	-	-	-	-	-	-

Supplementary Table 6. Residue polymorphism within the VS2 among different clades and subclades of H5N1

Virus strain	Clade	Mean ID_{50}	Residue at VS2																															
			130	131	132	133	134	135	136	137	153	154	155	156	157	158	159	160	161	186	187	188	189	190	191	192	193	194	222	223	224	225	226	227
A/duck/Guangxi/1378/2004	5	16631	E	A	S	S	G	V	S	S	W	L	I	K	K	N	S	A	Y	N	D	A	A	E	Q	T	K	L	K	V	N	G	Q	S
A/chicken/Henan/16/2004	8	14414	-	-	-	-	-	-	-	-	-	-	-	-	-	-	-	T	-	-	-	-	-	-	-	-	R	-	-	-	-	-	-	-
A/goose/Shantou/1621/2005	9	10724	-	-	-	-	-	-	-	-	-	-	-	-	-	-	-	-	-	-	-	-	-	-	-	-	-	-	-	-	-	-	-	-
A/silky chicken/Hong Kong/SF189/2001	3	10597	-	-	-	-	-	-	-	-	-	-	-	-	-	-	-	T	-	-	-	-	-	-	-	-	-	-	-	-	-	-	-	-
A/Beijing/01/2003	7	7864	-	-	-	-	-	-	-	-	-	-	-	-	-	-	N	T	-	-	-	E	-	-	-	I	-	-	-	-	-	-	-	-
A/blackbird/Hunan/1/2004	6	7256	-	-	-	-	-	-	-	-	-	-	-	-	-	-	-	-	-	-	-	-	-	-	-	-	-	-	-	-	-	-	-	-
A/Turkey/65596/2006	2.2.1	5580	-	-	-	-	-	-	-	-	-	-	-	-	-	D	N	-	-	-	-	-	-	-	-	-	R	-	-	-	-	-	-	N
A/Indonesia/5/2005	2.1.3.2	5175	-	-	-	-	-	-	-	-	-	-	-	-	-	-	-	T	-	-	-	-	-	-	-	-	R	-	-	-	-	-	-	-
A/Shenzhen/406H/2006	2.3.4	4953	-	-	-	-	-	-	-	-	-	-	-	-	-	-	N	T	-	-	-	-	-	-	-	-	-	-	-	-	-	-	-	-
A/chicken/Korea/es/2003	2.5	4640	-	-	-	-	-	-	-	-	-	-	-	-	-	-	-	-	-	-	-	-	-	-	-	-	R	-	-	-	-	-	-	-
A/Thailand/(KAN-1)/2004	1	4094	-	-	-	L	-	-	-	-	-	-	-	-	-	-	-	T	-	-	-	-	-	-	-	-	-	-	-	-	-	-	-	-
A/chicken/Guangxi/12/2004	2.4	2578	-	-	-	-	-	-	-	-	-	-	-	-	-	-	-	-	-	-	-	-	-	-	-	-	R	-	-	-	-	-	-	-
A/Chicken/Vietnam /NCVD-16/08	7.1	1986	-	-	-	-	-	-	-	A	-	-	-	-	-	-	N	T	-	-	-	E	K	-	-	I	R	I	-	-	-	-	-	-
A/Hong Kong/156/1997	0	1933	D	-	-	-	-	-	-	-	-	-	-	-	-	-	-	-	-	-	-	-	-	-	-	-	-	-	-	-	-	-	-	-
A/Chicken/Vietnam/NCVD-03/08	7.1	1814	-	-	-	-	-	-	-	A	-	-	-	-	-	-	N	T	-	-	N	E	-	-	-	I	Q	I	-	-	-	-	-	-
A/goose/Guiyang/337/2006	4	609	-	-	-	L	-	-	-	-	-	-	-	-	-	-	-	S	-	-	-	-	-	-	-	I	-	-	-	-	-	-	-	-
A/common magpie/Hong Kong/5052/2007	2.3.2.1	192	-	-	-	L	-	-	-	-	-	-	-	-	-	G	N	-	-	-	-	E	-	-	-	-	R	-	-	-	-	-	-	-

Supplementary Table 7. Residue polymorphism within the VS3 among different clades and subclades of H5N1

Virus strain	Clade	Mean ID_{50}	Residue at VS3																						
			77	78	79	80	81	81a	82	83	117	118	119	120	121	140	141	142	143	144	145	146	147	148	149
A/duck/Guangxi/1378/2004	5	16631	D	E	F	I	N	V	P	E	H	F	E	K	I	P	Y	L	G	R	P	S	F	F	R
A/chicken/Henan/16/2004	8	14414	-	-	-	-	-	-	-	-	-	-	-	-	-	-	-	Q	-	K	S	-	-	-	-
A/goose/Shantou/1621/2005	9	10724	-	-	-	-	-	-	-	-	-	-	-	-	-	-	-	-	-	K	-	-	-	-	-
A/silky chicken/Hong Kong/SF189/2001	3	10597	-	-	-	-	-	-	-	-	-	-	-	-	-	-	-	-	-	K	S	-	-	-	-
A/Beijing/01/2003	7	7864	-	-	-	-	-	-	-	-	-	-	-	-	-	-	-	-	-	-	-	-	-	-	-
A/blackbird/Hunan/1/2004	6	7256	-	-	-	-	-	-	-	-	-	-	-	-	-	-	-	Q	-	N	-	-	-	-	-
A/Turkey/65596/2006	2.2.1	5580	-	-	-	L	-	-	-	-	-	-	-	-	-	-	-	Q	-	-	S	-	-	-	-
A/Indonesia/5/2005	2.1.3.2	5175	-	-	-	-	-	-	-	-	-	-	-	-	-	-	-	-	-	S	-	-	-	-	-
A/Shenzhen/406H/2006	2.3.4	4953	-	-	-	-	-	-	-	-	-	-	-	-	-	-	-	Q	-	T	-	-	-	-	-
A/chicken/Korea/es/2003	2.5	4640	-	-	-	-	-	-	-	-	-	-	-	-	-	-	-	Q	-	-	S	-	-	-	-
A/Thailand/(KAN-1)/2004	1	4094	-	-	-	-	-	-	-	-	-	-	-	-	-	-	-	Q	R	K	S	-	-	-	-
A/chicken/Guangxi/12/2004	2.4	2578	-	-	-	-	-	-	-	-	-	-	-	-	-	-	-	Q	-	K	S	-	-	-	-
A/Chicken/Vietnam /NCVD-16/08	7.1	1986	-	-	-	L	-	-	S	-	-	L	K	-	-	S	-	-	-	E	-	-	-	-	-
A/Hong Kong/156/1997	0	1933	-	-	-	-	-	-	-	-	-	-	-	-	-	-	-	-	-	-	S	-	-	-	-
A/Chicken/Vietnam/NCVD-03/08	7.1	1814	-	-	-	L	-	-	S	-	-	L	K	-	-	S	-	M	-	E	-	-	-	-	-
A/goose/Guiyang/337/2006	4	609	-	-	-	-	-	-	-	-	-	-	-	-	-	-	-	-	-	E	S	-	-	-	-
A/common magpie/Hong Kong/5052/2007	2.3.2.1	192	-	-	-	-	-	-	-	-	-	-	-	-	-	-	-	Q	-	N	S	-	-	-	-

Supplementary Table 8. Residue polymorphism within the VS4 among different clades and subclades of H5N1

Virus strain	Clade	Mean ID_{50}	Residue at VS4																			
			53	54	55	55a	56	57	58	59	60	61	62	272	273	274	275	276	277	278	279	280
A/duck/Guangxi/1378/2004	5	16631	D	L	D	G	V	K	P	L	I	L	R	L	E	Y	G	N	C	N	T	K
A/chicken/Henan/16/2004	8	14414	-	-	-	-	-	-	-	-	-	-	-	-	-	-	-	-	-	-	-	-
A/goose/Shantou/1621/2005	9	10724	-	-	-	-	-	-	-	-	-	-	-	-	-	-	-	-	-	-	-	-
A/silky chicken/Hong Kong/SF189/2001	3	10597	-	-	-	-	-	-	-	-	-	-	-	-	-	-	-	-	-	-	-	-
A/Beijing/01/2003	7	7864	-	-	-	-	-	-	-	-	-	-	-	-	-	-	-	-	-	-	-	-
A/blackbird/Hunan/1/2004	6	7256	E	-	-	-	-	-	-	-	-	-	-	-	-	-	-	-	-	-	-	-
A/Turkey/65596/2006	2.2.1	5580	-	-	-	-	-	-	-	-	-	-	-	-	-	-	-	-	-	-	-	-
A/Indonesia/5/2005	2.1.3.2	5175	-	-	-	-	-	-	-	-	-	-	-	-	-	-	-	-	-	-	-	-
A/Shenzhen/406H/2006	2.3.4	4953	-	-	-	-	-	-	-	-	-	-	-	V	-	-	-	-	-	-	-	-
A/chicken/Korea/es/2003	2.5	4640	-	-	-	-	-	-	-	-	-	-	-	-	-	-	-	-	-	-	-	-
A/Thailand/(KAN-1)/2004	1	4094	-	-	-	-	-	-	-	-	-	-	-	-	-	-	-	-	-	-	-	-
A/chicken/Guangxi/12/2004	2.4	2578	-	-	-	-	-	-	-	-	-	-	K	-	-	-	-	D	-	-	-	-
A/Chicken/Vietnam /NCVD-16/08	7.1	1986	N	-	-	-	-	-	-	-	-	-	K	-	-	-	-	-	-	-	-	-
A/Hong Kong/156/1997	0	1933	-	-	N	-	-	-	-	-	-	-	-	-	-	-	-	-	-	-	-	-
A/Chicken/Vietnam/NCVD-03/08	7.1	1814	N	-	-	-	-	-	-	-	-	-	K	-	-	-	-	-	-	-	-	-
A/goose/Guiyang/337/2006	4	609	S	-	-	-	-	-	-	-	-	-	-	-	-	-	-	-	-	-	-	-
A/common magpie/Hong Kong/5052/2007	2.3.2.1	192	-	-	N	-	-	-	-	-	-	-	K	V	-	-	-	-	-	-	-	R

Supplementary References

1. Khurana, S., *et al.* Antigenic fingerprinting of H5N1 avian influenza using convalescent sera and monoclonal antibodies reveals potential vaccine and diagnostic targets. *PLoS Med* **6**, e1000049 (2009).
2. Philpott, M., Hioe, C., Sheerar, M. & Hinshaw, V.S. Hemagglutinin mutations related to attenuation and altered cell tropism of a virulent avian influenza A virus. *J Virol* **64**, 2941-2947 (1990).
3. Kaverin, N.V., *et al.* Structure of antigenic sites on the haemagglutinin molecule of H5 avian influenza virus and phenotypic variation of escape mutants. *J Gen Virol* **83**, 2497-2505 (2002).
4. Kaverin, N.V., *et al.* Epitope mapping of the hemagglutinin molecule of a highly pathogenic H5N1 influenza virus by using monoclonal antibodies. *J Virol* **81**, 12911-12917 (2007).
5. Wu, W.L., *et al.* Antigenic profile of avian H5N1 viruses in Asia from 2002 to 2007. *J Virol* **82**, 1798-1807 (2008).
6. Li, J., *et al.* Fine antigenic variation within H5N1 influenza virus hemagglutinin's antigenic sites defined by yeast cell surface display. *Eur J Immunol* **39**, 3498-3510 (2009).
7. Chen, Y., *et al.* Broad cross-protection against H5N1 avian influenza virus infection by means of monoclonal antibodies that map to conserved viral epitopes. *J Infect Dis* **199**, 49-58 (2009).
8. Ho, H.T., *et al.* Rapid detection of H5N1 subtype influenza viruses by antigen capture enzyme-linked immunosorbent assay using H5- and N1-specific monoclonal antibodies. *Clin Vaccine Immunol* **16**, 726-732 (2009).
9. Yoshida, R., *et al.* Cross-protective potential of a novel monoclonal antibody directed against antigenic site B of the hemagglutinin of influenza A viruses. *PLoS Pathog* **5**, e1000350 (2009).
10. Prabhu, N., *et al.* Prophylactic and therapeutic efficacy of a chimeric monoclonal antibody specific for H5 haemagglutinin against lethal H5N1 influenza. *Antivir Ther* **14**, 911-921 (2009).
11. Rudneva, I.A., *et al.* Antigenic epitopes in the hemagglutinin of Qinghai-type influenza H5N1 virus. *Viral Immunol* **23**, 181-187 (2010).
12. Prabakaran, M., *et al.* Neutralizing epitopes of influenza virus hemagglutinin: target for the development of a universal vaccine against H5N1 lineages. *J Virol* **84**, 11822-11830 (2010).
13. Han, T., *et al.* Fine epitope mapping of monoclonal antibodies against hemagglutinin of a highly pathogenic H5N1 influenza virus using yeast surface display. *Biochem Biophys Res Commun* **409**, 253-259 (2011).
14. Ohkura, T., *et al.* Epitope mapping of neutralizing monoclonal antibody in avian influenza A H5N1 virus hemagglutinin. *Biochem Biophys Res Commun* **418**, 38-43 (2012).
15. Du, L., *et al.* Identification and structural characterization of a broadly neutralizing antibody targeting a novel conserved epitope on the influenza virus H5N1 hemagglutinin. *J Virol* **87**, 2215-2225 (2013).
